# Village-Randomized Clinical Trial of Home Distribution of Zinc for Treatment of Childhood Diarrhea in Rural Western Kenya

**DOI:** 10.1371/journal.pone.0094436

**Published:** 2014-05-16

**Authors:** Daniel R. Feikin, Godfrey Bigogo, Allan Audi, Sherri L. Pals, George Aol, Charles Mbakaya, John Williamson, Robert F. Breiman, Charles P. Larson

**Affiliations:** 1 International Emerging Infections Program, Centers for Disease Control and Prevention Kisumu, Kenya; 2 Division of Global HIV/AIDS, United States Centers for Disease Control and Prevention, Atlanta, Georgia, United States of America; 3 Center for Global Health, United States Centers for Disease Control and Prevention, Atlanta, Georgia, United States of America; 4 Kenya Medical Research Institute, Nairobi, Kenya; 5 International Centre for Diarrhoeal Disease Research, Bangladesh, Dhaka, Bangladesh; 6 Department of Pediatrics, University of British Columbia, Vancouver, Canada; The George Washington University Medical Center, United States of America

## Abstract

**Background:**

Zinc treatment shortens diarrhea episodes and can prevent future episodes. In rural Africa, most children with diarrhea are not brought to health facilities. In a village-randomized trial in rural Kenya, we assessed if zinc treatment might have a community-level preventive effect on diarrhea incidence if available at home versus only at health facilities.

**Methods:**

We randomized 16 Kenyan villages (1,903 eligible children) to receive a 10-day course of zinc and two oral rehydration solution (ORS) sachets every two months at home and 17 villages (2,241 eligible children) to receive ORS at home, but zinc at the health–facility only. Children’s caretakers were educated in zinc/ORS use by village workers, both unblinded to intervention arm. We evaluated whether incidence of diarrhea and acute lower respiratory illness (ALRI) reported at biweekly home visits and presenting to clinic were lower in zinc villages, using poisson regression adjusting for baseline disease rates, distance to clinic, and children’s age.

**Results:**

There were no differences between village groups in diarrhea incidence either reported at the home or presenting to clinic. In zinc villages (1,440 children analyzed), 61.2% of diarrheal episodes were treated with zinc, compared to 5.4% in comparison villages (1,584 children analyzed, p<0.0001). There were no differences in ORS use between zinc (59.6%) and comparison villages (58.8%). Among children with fever or cough without diarrhea, zinc use was low (<0.5%). There was a lower incidence of reported ALRI in zinc villages (adjusted RR 0.68, 95% CI 0.46–0.99), but not presenting at clinic.

**Conclusions:**

In this study, home zinc use to treat diarrhea did not decrease disease rates in the community. However, with proper training, availability of zinc at home could lead to more episodes of pediatric diarrhea being treated with zinc in parts of rural Africa where healthcare utilization is low.

**Trial Registration:**

ClinicalTrials.gov NCT00530829

## Introduction

Diarrhea caused approximately 700,000 deaths in children in 2011, mostly in countries where children are deficient in zinc, an essential trace element for optimal immune response to infections. [1]–[4] Zinc treatment of a diarrheal episode has been shown to decrease the duration and lessen the severity of that diarrhea episode. [1], In addition, zinc given for 10–14 days during or shortly after a diarrheal episode has been shown to decrease subsequent diarrhea and acute lower respiratory illness infections over the ensuing few months. [2], [7], [8] Based on the weight of available evidence, WHO and UNICEF recommend zinc as an adjunct for diarrhea treatment for children in developing countries, and promote zinc use in the recently published integrated Global Action Plan for Pneumonia and Diarrhoea (GAPPD). [6], [9].

Scaling up the use of zinc in developing countries has been challenging, due to obstacles of supply, cost, distribution and training. [10], [11] Within the public sector, the initial push has been to make zinc available in health-facilities. Yet, many children with diarrhea in developing countries never present to public sector health-facilities, due to constraints of time, distance and cost of transport. [12], [13] Distribution of zinc through community health workers (CHWs) has had positive results in several sites in South Asia and Africa. [7], [8], [14] There are parts of Africa, though, where CHWs do not treat illness in the community. Given the evidence of zinc’s prevention of diarrhea and respiratory illness, we investigated whether providing zinc at home for future treatment of diarrhea had a preventative impact at the community level on the reduction of diarrhea incidence. As secondary objectives, we evaluated if availability of zinc in the home had a preventative impact at the community level on the reduction of respiratory disease incidence and would result in more diarrhea episodes being treated with zinc than having zinc available in the clinic only. We used a village-randomized design to facilitate training, distribution, and equity among caretakers within a village.

## Methods

The protocol and consent forms were reviewed and approved by the Ethical Review Boards of KEMRI (# 932 and 985) and CDC (# 4566 and 4678). Written informed consent was obtained from children’s primary caretakers. The study was registered with ClinicalTrials.gov (#NCT00530829). The protocol for this trial and supporting CONSORT checklist are available as supporting information; see Checklist S1 and Protocol S1.

### Surveillance Platform

Population-based infectious disease surveillance (PBIDS) has been ongoing since late 2006 in Asembo, western Kenya. [15] On July 1, 2008, PBIDS included 3,856 children <5 years of age, in 33 villages. Malaria transmission is endemic and occurs year-round. [16] The under-5 mortality ratio was 212 per 1,000 live births in 2008. [17].

The methods for PBIDS have been described. [15], [18] In brief, community interviewers visit enrolled households every two weeks to inquire about illnesses, referred to as household morbidity surveillance (HMS). For key symptoms, including cough, fever and diarrhea, participants provide number of days during the past two weeks when they had those symptoms. Health-care seeking, hospital admission, and medication use are also documented. For children <5 years, the mother, or other knowledgeable caretaker, is interviewed. Abbreviated physical exams are done.

In Lwak Hospital, the centrally-located referral facility for PBIDS, medical care is free to PBIDS participants. Patients are examined and diagnosed by clinical officers (similar to physician’s assistants). Malaria blood smears are done on all febrile patients. Structured questionnaires record symptoms, vital signs, physical exam findings, diagnosis, and treatment. All children with diarrhea presenting to Lwak Hospital during the study were given zinc, if they were not already taking it for the episode, as standard protocol. We have previously shown that of children in PBIDS with diarrhea who are brought to a clinic, half are brought to Lwak Hospital. [19].

### Zinc Study Design

The primary objective was to assess if home zinc availability led to decreased diarrhea incidence in the community. Secondary objectives were if home zinc availability led to decreased respiratory infections in the community, and if more children with diarrhea were treated with zinc when available in the home. For the zinc study, all residents in the 33 PBIDS villages aged 2–59 months of age on December 1, 2007 were identified. Among these, only participants enrolled in PBIDS and found at home after three attempts were eligible for enrollment. Enrollment was rolling until January 2009, whereby children born after the start of the zinc study or who in-migrated were eligible after they reached 2 months of age, whereas children reaching their 5^th^ birthday were excluded. The 33 villages were randomly assigned to receive a 10-day blister pack of 20 mg dispersible zinc-sulfate tablets (Nutriset, France) at home every two months (hereafter referred to as “zinc villages”) or to receive no zinc at home, but were told they could obtain it at Lwak Hospital if their child had diarrhea (hereafter referred to as “comparison villages”). All study participants, regardless of village category, presenting to Lwak clinic with diarrhea received a 10-day blister pack of zinc, if they were not already taking it for that episode. Every two months, households of all participants, regardless of study arm, received 2 packets of low-osmolarity ORS. All medicines were given free of charge.

Distribution of home zinc/ORS was done by village reporters, women who lived in the village and had minimal health-related training. Village reporters received a week-long training initially and refresher trainings every 2 months on treatment of diarrhea in children, how and when to use zinc and ORS, when mothers should take their child to the clinic, and were instructed to emphasize that zinc and/or ORS use was not a substitute for taking their child to the clinic. Special emphasis was given in training the village reporters that zinc was to treat only diarrhea, and if a child had other symptoms, such as fever or cough, they might also need an antimalarial or antibiotic at the clinic. [20] At each bi-monthly home visit, the village reporters re-educated mothers on proper use of zinc and ORS. Village reporters, who were unblinded to study arm, delivered zinc and/or OR were every 2 months, regardless of whether the previous supply had been used.

Although part of the MOH essential drug list, zinc was not available in MOH-run health facilities at the start of this study in February 2008. Nor was zinc available in the private sector in rural Kenya at that time. Therefore, zinc was only available to children with diarrhea presenting to Lwak Hospital. By late 2008, zinc had started to become available in MOH-run health facilities in the study area, although stock-outs were frequent.

Participants who had a diarrhea episode reported at the regular bi-weekly HMS visits were visited again seven days later to inquire about ongoing illness, medication use, and any adverse events.

### Laboratory

To establish baseline zinc levels in the study area we randomly selected eight children from each of 33 villages, with the intent to enroll the first five who were available and consented for each village, for a goal of 165 children. We measured serum zinc concentrations, using Flame Atomic Absorption Spectrophotometry, at the KEMRI laboratory in Nairobi. Calibration of instruments was done using sera with known concentrations provided by ICDDR-B in Bangladesh. Zinc deficiency in children was considered as a concentration <65 mg/dl. [3], [21].

### Case Definitions

Case definitions for syndromes for HMS and clinic surveillance are given in Table 1. [15] Definitions for diarrhea, dehydration and ALRI follow WHO’s Integrated Management of Childhood Illness guidelines. [22].

**Table 1:** Case definitions for major infectious disease syndromes from clinic and household morbidity surveillance (HMS) in Asembo, western Kenya.

Syndrome	Clinic definition	Household definition
Acute respiratoryinfection (ARI)	(≥1 symptom): cough, difficulty breathing, chest pain, sore throat, sneezing, ear complaintsor runny nose	cough or difficulty breathing
Acute LowerRespiratoryInfection (ALRI)	cough or difficulty breathing with one of the following: elevated respiratory rate for age*(non-severe pneumonia;) or IMCI danger sign†, lower chest wall indrawing, stridor, or oxygensaturation <90% (severe/very severe pneumonia)	cough or difficulty breathing and rapid respiration for age* or chest indrawing noted on exam
Diarrhea	≥3 looser than normal stools in a 24 hour period. Severe defined as IMCI danger sign orsymptom/sign dehydration‡	≥3 looser than normal stools in a 24 hour period
Acute FebrileIllness (AFI)	documented axillary temperature ≥38.0°C without an obvious cause, defined as cough,difficulty breathing, chest pain, signs of meningitis, or bloody diarrhea (positivemalaria smear is not an exclusion)	report of fever, without evidence of another infection defined as cough or difficulty breathing or bloody diarrhea

*Elevated respiratory rate for age based on WHO Integrated Management of Childhood Illness algorithm [14]; <2 months, ≥60 breaths/minute; 2–11 months, ≥50 breaths/minute; 12–59 months, ≥40 breaths/minute.

† IMCI danger signs are maternal report of convulsions, inability to drink or breastfeed, or vomiting everything, or on exam lethargy or unconsciousness [22].

‡ IMCI signs/symptoms of dehydration are the following: sunken eyes, slow skin pinch, restless/irritable behavior, drinking eagerly or not at all [22].

### Village Randomization

We chose a randomization method that would probabilistically maintain similar numbers of children in each village category, by alternating village assignment, after a random starting assignment, based on an ordered list of villages by population size. Villages were not matched or stratified based on baseline rates of morbidity, mortality or health-seeking patterns. Village randomization was done at KEMRI/CDC after consenting all initial participants in December 2007. Village reporters informed participants of their village group at the time of first home zinc/ORS distribution in January 2008. Mothers from comparison villages could not be given zinc by village reporters since their group assignment was by their village of residence; all village reporters were from the villages and knew mothers in their own village.

### Data Analysis

We considered the first 2 months (January–February 2008) a pilot study, excluding those months from analysis. Therefore, the study period for analysis was February 2008 to March 2009. We considered the pre-intervention period as October 2006–November 2007, which was when HMS data were available for all villages. Pre-intervention period data were included in analyses to account for baseline differences in disease rates and distance to clinic between village categories. Pre-intervention data on medication use were not available.

Because the population of children in the PBIDS villages was fixed, we calculated what decrease in diarrhea incidence we could detect with a power of 80% or greater at an alpha of 0.05. We estimated the PBIDS population of children 2–59 months old to be approximately 3,000, and assumed a baseline diarrhea incidence of 3.6 episodes per year. Assuming that there would be a 5% decrease in diarrhea rates in the comparison villages during the study period due to zinc use in the clinic, we estimated that we would have the power to detect a difference of 12% in rates between zinc and comparison groups, accounting for correlation of diarrhea events by child using the method described by Rochon. [23].

Baseline characteristics between children in zinc and comparison villages were compared by chi-square or t-test, controlling for clustering at the village level. The percentages of episodes of illness in the zinc and comparison villages that resulted in zinc or other medication use, or care-seeking outside the home, were compared using the F-test in SAS PROC GLIMMIX with village as a random effect to control for clustering; for the analysis of care-seeking we adjusted for seeking care outside the home for similar illness episodes in the pre-intervention period. Rates of illness were compared between children in zinc and comparison villages, both for those resulting in a sick visit to Lwak Hospital and those reported during the biweekly HMS visits. For the HMS, rates were calculated using newly reported episodes divided by the number of total days from which HMS data were available; due to poor recall of symptoms we only calculated rates for the day of the home visit and the three previous days, as we have described before. [15], [24] For the clinic, rates were calculated by using the number of clinic visits for a syndrome divided by the person-time of residence in the surveillance area. Children contributed person-time from the date of their enrollment until the end of the study (March 31, 2009), their fifth birthday, or their date of outmigration or death. Rate ratios were calculated between zinc and comparison villages, adjusting for pre-intervention rates of the syndrome in the child’s village, distance of the child’s compound to Lwak Hospital, and the child’s age. For pre-intervention rates, all children in the study villages aged 2–59 months enrolled in morbidity surveillance were used, whereas rates during the intervention period were limited to only those enrolled in the zinc study. Analyses of rates were done using poisson regression with generalized estimating equations to control for correlated observations (PROC GENMOD, SAS version 9.1). A second “per-treatment” analysis was done for clinic visits, where we included only children who took at least one course of zinc (intervention villages) or ORS (comparison villages) during the course of the study. All data analysis was done unblinded to study arm.

## Findings

**Figure 1:**
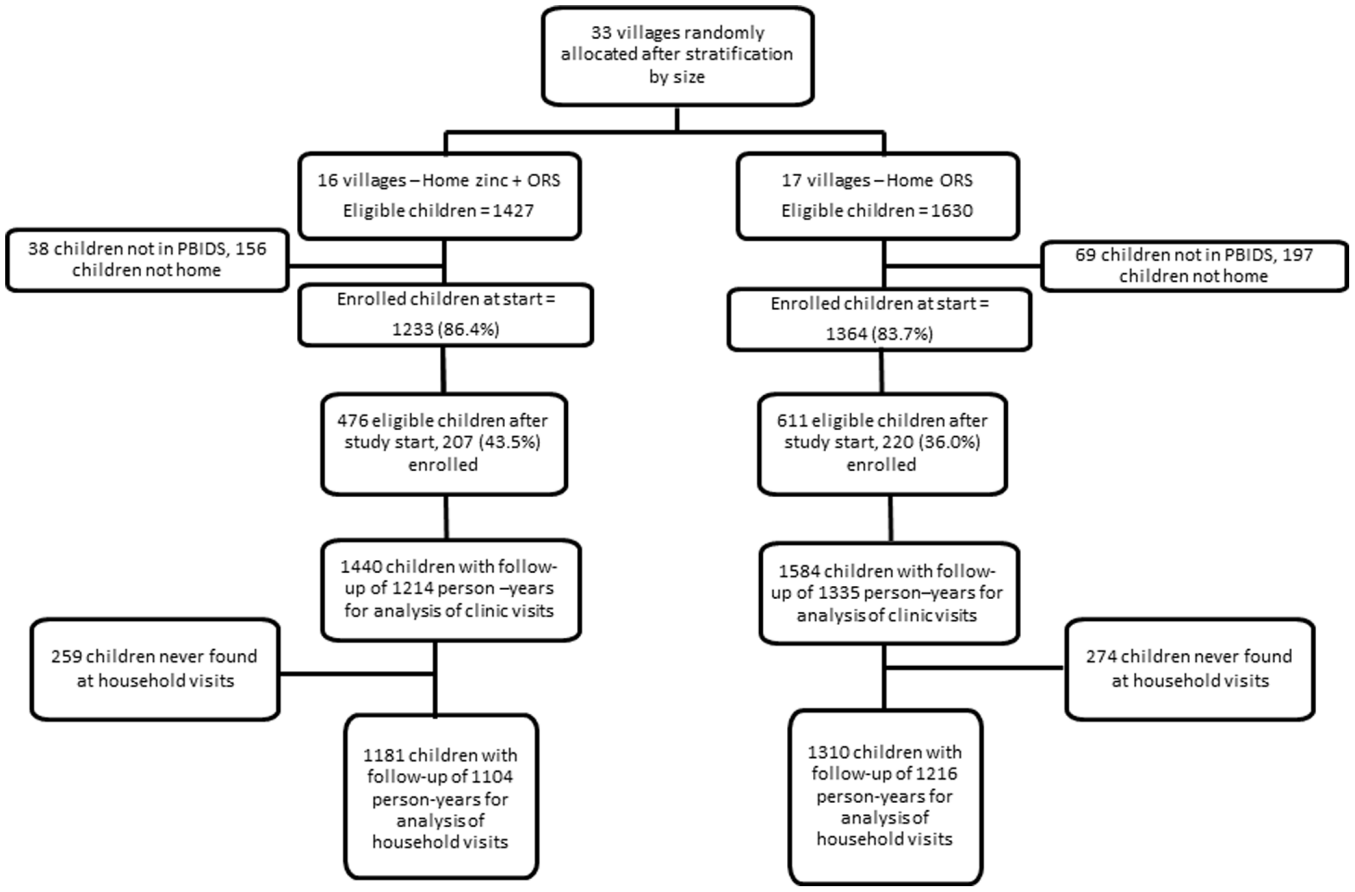
Trial profile. Village randomization and number of children enrolled at the initial enrollment in December 2007 and during the ongoing enrollment during the study period from February 2008–March 2009. Children and person-time contribution given for the analysis of clinic-based surveillance and household morbidity surveillance – see methods. PBIDS = Population-based Infectious Disease Surveillance.

In total, 1,440 children were enrolled in 16 zinc villages and 1,584 in 17 comparison villages (Figure 1). Over 80% of eligible children were enrolled at the outset in both groups; however, for children who were born or in-migrated into the area after the start of the study, only 36–44% were enrolled due to delays in registering children into the surveillance system. Of enrolled children, all were included in the clinic-based analysis, but 17% were excluded from the household-based analysis since no household morbidity data was available for these children during the study period; this was likely due to nobody being at home during biweekly household visits or temporary relocation of residences. During the study period, in the zinc and comparison villages, 96 and 106 children moved out of the study area, 31 and 41 died, and 346 and 431 reached their fifth birthday, respectively.

Zinc and comparison villages were similar in terms of children’s age, maternal education, socioeconomic status, and crowding (Table 2). Zinc villages tended to be further from Lwak Hospital than comparison villages (4.7 vs. 3.4 kilometers, p = 0.07). At baseline, we collected blood for zinc concentration measurement from 151 children who were enrolled and consented. Of these, 135 (89%) blood samples of adequate volume and quality, the median zinc concentration was 72.0 µg/dL (IQR 61–88); 32% of children had low zinc levels (<65 µg/dL). There was no difference between median zinc concentrations of children in the zinc group (72 µg/dL) and comparison group (73.0 µg/dL, p = 0.87).

**Table 2:** Baseline characteristics for participants in home zinc and comparison villages, western Kenya, February 2008–March 2009.

Characteristic	Home zinc	Comparison	P value*
N	1440	1584	
Age in months (median)	28.2	27.9	0.58
Age categories, n (%)			
2–5 months	202 (14)	246 (16)	0.31
6–11 mo	139 (10)	147 (9)	
12–23 mo	278 (19)	279 (18)	
24–59 mo	821 (57)	912 (58)	
Male, n (%)	732 (51)	772 (49)	0.38
Maternal education beyond primary	291 (20)	322 (20)	0.89
Poorer households†	345 (29)	379 (28)	0.79
Distance from residence to Lwak (median meters)	4728	3351	0.07
# children <5 years in household (median)	2.0	2.0	0.36

*p value controls for cluster design by village.

† Poorer household is defined as the lowest two quintiles using multiple component analysis.

There were 969 7-day follow up visits for diarrheal episodes in zinc villages and 953 in comparison villages. In zinc villages 6.1% of children still had diarrhea versus 5.6% in comparison villages (p = 0.57). Among zinc users, the median number of days that zinc had been taken was 8.0 in both village groups. Twenty-one percent of children given zinc had vomited a zinc tablet; 69% of vomiting episodes were limited to the first zinc dose (no difference by village group). The median number of ORS sachets used was 2.0.

Based on self-report of the caretaker at the biweekly HMS visit, in zinc villages 61.2% of all diarrheal episodes were treated with zinc, compared to 5.4% in the comparison villages (p<0.0001, Table 3). Similar differences in zinc use were observed for episodes of diarrhea with fever and with cough. There were no differences in ORS use for diarrhea between zinc (59.6%) and comparison villages (58.8%). Diarrheal episodes in zinc villages resulted in less frequent use of antimalarials and antibiotics (Table 3). Children with diarrhea and reported fever in zinc villages (approximately two-thirds of diarrheal episodes) received an antimalarial less frequently (17.8%) than in comparison villages (23.5%, p = 0.048). Care-seeking outside the home for diarrhea episodes was similar between village groups. Among children with fever or cough without diarrhea, zinc use was low in both zinc and comparison villages (<0.5%), although care-seeking outside the home was higher in comparison villages for these non-diarrhea syndromes.

**Table 3:** Effect of intervention on drug use and healthcare use for various disease syndromes during the intervention period from household morbidity surveillance (HMS), western Kenya, February 2008–March 2009.

	Home zinc N (%†)	Comparison N (%†)	P value*
**All diarrhea (n)**	1707	1700	
Zinc	1045 (61.2)	92 (5.4)	<.0001¶
ORS	1018 (59.6)	999 (58.8)	.62
Antimalarial	190 (11.1)	272 (16.0)	.006
Antibiotic	291 (17.1)	383 (22.5)	.018
Sought care†	531 (31.2)	542 (31.9)	.22
**Diarrhea+fever (n)**	1043	1133	
Zinc	624 (59.8)	72 (6.4)	<.0001
ORS	629 (60.3)	655 (57.8)	.31
Antimalarial	186 (17.8)	266 (23.5)	.048
Antibiotic	253 (24.3)	322 (28.4)	.22
Sought care†	380 (36.5)	412 (36.4)	.26
**Diarrhea+cough (n)**	701	716	
Zinc	415 (59.2)	45 (6.3)	<.0001
ORS	426 (60.8)	400 (55.9)	.11
Antimalarial	122 (17.4)	150 (21.0)	.28
Antibiotic	192 (27.4)	226 (31.6)	.21
Sought care†	253 (36.1)	255 (35.7)	.61
**Fever without diarrhea (n)**	6188	6693	
Zinc	13 (0.2)	2 (0.03)	N/A
ORS	25 (0.4)	37 (0.6)	.33
Antimalarial	1510 (24.4)	1701 (25.4)	.81
Antibiotic	1187 (19.2)	1221 (18.2)	.70
Sought care†	2509 (40.6)	3023 (45.3)	.017
**Cough without diarrhea (n)**	5412	5170	
Zinc	10 (0.2)	1 (0.02)	.039
ORS	12 (0.2)	22 (0.4)	.31
Antimalarial	868 (16.0)	883 (17.1)	.78
Antibiotic	1396 (25.8)	1368 (26.5)	.48
Sought care†	1999 (36.9)	2089 (40.5)	.019

*p-value calculation includes a random effect variable for village, and adjusts for pre-intervention rates of seeking care outside the home by village - F-test in SAS PROC GLIMMIX.

† % given in table is the % of biweekly household visits with illness that resulted in medication use or care-seeking outside the home. Denominator for sought care was slightly lower than for medication use due to some missing data for that variable.

¶ For primary outcome, intracluster correlation (ICC) = 0.030.

Although rates of clinic visitation due to diarrhea were lower in the zinc than comparison villages during the intervention period, this difference was not statistically different after adjustment (Table 4). Similarly, no significant differences were observed between zinc and comparison villages for clinic visits for rates of severe diarrhea, ARI, ALRI, acute febrile illness or malaria parasitemia (Table 4). We found similar results when limiting the clinic-based analysis to only those children who took at least one course of zinc or ORS during the study period (Table 5). There were also no significant differences in the incidence of reported diarrhea, diarrhea with fever, severe diarrhea, ARI, or acute febrile illness at the biweekly household visits (Table 6). However, children in the zinc villages had 32% fewer reported ALRI episodes than in comparison villages (adjusted RR 0.68, 95% CI 0.46–0.99). There were no differences between village groups in hospitalization rates or all-cause mortality (Table 4).

**Table 4:** Effect of home zinc on rate of sick visits to Lwak clinic, hospitalization, and mortality, western Kenya, from February 2008–March 2009, controlling for baseline rates of morbidity in home zinc and comparison villages.

	Pre-intervention*		Intervention period†		Adjusted RR‡ (CI 95%)
	Home zinc	Comparison	Home zinc	Comparison	
Person-years observed	2001	2245	1214	1335	
Diarrhea	0.098	0.118	0.146	0.184	1.05 [0.81–1.37]¶
Diarrhea plus reported fever	0.089	0.102	0.138	0.160	1.09 [0.83–1.43]
Diarrhea and malaria	0.025	0.023	0.072	0.090	1.05 [0.74–1.49]
Severe diarrhea	0.040	0.046	0.059	0.059	1.17 [0.79–1.76]
Diarrhea hospitalization	0.027	0.034	0.037	0.051	0.87 [0.56–1.36)
Acute Respiratory Illness	0.406	0.464	0.830	0.904	1.16 [0.98–1.37]
ALRI	0.053	0.065	0.083	0.095	1.06 [0.78–1.45]
ALRI hospitalization	0.035	0.049	0.041	0.055	0.91 [0.59–1.39]
Acute febrile illness	0.030	0.049	0.145	0.162	1.30 [0.99–1.69]
Malaria (Blood smear+)	0.033	0.040	0.530	0.611	1.19 [0.99–1.44]
All-cause hospitalization	0.093	0.126	0.161	0.195	1.06 [0.82–1.38]
Mortality	0.023	0.029	0.024	0.029	0.83 [0.49–1.40]

Intention-to-treat analysis includes all enrolled children. Rates are given as episodes per person-year.

*Pre-intervention period was October 2006 to November 2007.

† While pre-intervention includes all children in the village of appropriate age, the intervention period only includes those enrolled in the home zinc study.

‡ Rate ratio is comparing rates between home zinc and comparison groups during the intervention period, adjusted for pre-intervention rates of same syndrome in the child’s village, distance of child’s compound to Lwak Hospital and child’s age.

¶ For primary outcome, intracluster correlation (ICC) = 0.027.

**Table 5:** Effect of home zinc on rate of sick visits to Lwak clinic, hospitalization, and mortality, western Kenya, from February 2008–March 2009, controlling for baseline rates of morbidity in home zinc and comparison villages.

	Pre-intervention*		Intervention period†		Adjusted RR‡ (CI 95%)
	Home zinc	Comparison	Home zinc	Comparison	
Person-years observed	2001	2245	598	625	
Diarrhea	0.098	0.118	0.22	0.31	0.99 [0.73–1.33]
Diarrhea plus reported fever	0.089	0.102	0.21	0.27	1.02 [0.76–1.39]
Diarrhea and malaria	0.025	0.023	0.10	0.14	0.87 [0.57–1.34]
Severe diarrhea	0.040	0.046	0.10	0.11	1.14 [0.72–1.79]
Diarrhea hospitalization	0.027	0.034	0.06	0.09	0.82 [0.49–1.36)
Acute Respiratory Illness	0.406	0.464	0.86	1.18	0.93 [0.76–1.13]
ALRI	0.053	0.065	0.11	0.15	0.95 [0.65–1.40]
ALRI hospitalization	0.035	0.049	0.06	0.09	0.93 [0.56–1.53]
Acute febrile illness	0.030	0.049	0.16	0.22	1.20 [0.84–1.71]
Malaria (Blood smear+)	0.033	0.040	0.49	0.77	0.89 [0.70–1.12]
All-cause hospitalization	0.093	0.126	0.17	0.29	0.79 [0.57–1.09]
Mortality	0.023	0.029	0.02	0.04	0.69 [0.34–1.41]

Per-treatment analysis includes only children who received at least one course of zinc in home zinc villages and one course of ORS in comparison villages. Rates are given as episodes per person-year.

*Pre-intervention period was October 2006 to November 2007.

† While pre-intervention includes all children in the village of appropriate age, the intervention period only includes those enrolled in the home zinc study who took one course of recommended treatment for diarrhea.

‡ Rate ratio is comparing rates between home zinc and comparison groups during the intervention period, adjusted for pre-intervention rates of same syndrome in the child’s village, distance of child’s compound to Lwak Hospital and child’s age.

**Table 6:** Effect of home zinc on rates of reported morbidity at the household morbidity surveillance (HMS), western Kenya, from February 2008–March 2009, controlling for baseline rates of morbidity in home zinc and comparison villages.

	Pre-intervention*		Intervention†		Adjusted RR‡ (CI 95%)
	Intervention	Comparison	Intervention	Comparison	
Person-years observed	1488	1690	1104	1206	
Diarrhea	2.64	1.69	1.94	1.99	0.96 (0.79–1.15)
Diarrhea plus reported fever	0.94	0.87	1.04	1.10	0.94 (0.81–1.24)
Severe diarrhea	1.90	1.21	1.15	1.32	0.91 (0.73–1.13)
Diarrhea hospitalization (any hospital)	0.04	0.04	0.05	0.06	0.97 (0.42–2.23)
Acute Respiratory Illness	7.32	6.78	7.26	6.83	1.03 (0.95–1.12)
ALRI	0.28	0.24	0.18	0.28	0.68 (0.46–0.99)
Acute febrile illness	10.06	10.13	10.16	10.36	0.99 (0.91–1.07)

Rates are given as episodes per person-year.

*Pre-intervention period was October 2006 to November 2007.

† While pre-intervention includes all children in the village of appropriate age, the intervention period only includes those enrolled in the home zinc study.

‡ Rate ratio is comparing rates between home zinc and comparison groups during the intervention period, adjusted for pre-intervention rates of the same syndrome in the child’s village, distance of village to Lwak and child’s age.

## Discussion

We did not observe a community-level impact on diarrhea incidence in home zinc villages despite increased zinc use. There are several potential reasons. First, it is possible that zinc use does not prevent future diarrhea episodes in this setting; most studies on the preventive effect of zinc on diarrhea occurred in South Asia and the epidemiology of diarrhea disease differs in Africa. [2], [7], [8], [25] Second, perhaps not enough children received zinc in the community to precipitate a measureable decrease in community-level incidence. We were not able to enroll as many children as we anticipated, particularly for the household-level morbidity analysis, and so it is possible we were unpowered to detect a difference. We did not, however, see reduced diarrhea rates when restricting the analysis to only children who took zinc. Third, possibly we were unable to detect a real decrease in diarrhea incidence due to limitations in our study design (e.g. baseline differences between village groups), which we were unable to fully adjust for. Moreover, biases in surveillance could have masked an impact on incidence; children given zinc at home might have had reduced health-seeking at clinic because of less perceived need for medication, or caretakers who gave zinc might have been more likely to recall and report diarrheal episodes at the home visits. Although we did not observe a difference in diarrhea rates, we did see marginally less ALRI in the zinc villages reported at the household, but not at the clinic; zinc treatment for diarrhea has been shown to prevent subsequent ALRI in India. [8].

While this study did not provide evidence for a community-level preventative impact of home zinc use, we did show that by providing zinc in the home children with diarrhea were much more likely to receive zinc treatment than when zinc was only available in the health facility. Moreover, having ORS available at home likely resulted in higher uptake of ORS for diarrhea treatment than occurred historically in this area. [9], [26], [27] In much of rural Africa, such as western Kenya, most children with diarrhea are not taken to health facilities. [12] We have previously shown in our area that only 36% of children with diarrhea seek care at a health facility. [26] Of note, an additional 50% of children sought care at sources besides licensed health facilities, suggesting that making zinc available through drug sellers, community health workers, or private providers could also increase the number of diarrheal episodes treated with zinc.

Our study demonstrated that caretakers of children can be educated to use zinc appropriately at home. In our study, very few caretakers used zinc to treat illnesses without diarrhea. Misuse of zinc has been raised as a concern whereby mothers might substitute one drug, particularly a free one, for other necessary drugs, like antimalarials, which have an extra real or opportunity cost to acquire. [20] Yet, our study did show some potential cautionary information similar to what was shown in a community-based zinc program in Mali in that children with diarrhea with fever tended to be less likely to receive antimalarial medications. [20] Although it is possible that fever could be part of the diarrhea episode, in an endemic malaria area, such as rural western Kenya, all episodes of fever merit either empiric malaria treatment or a reliable diagnostic test to rule out malaria. [28] This speaks to the need for ongoing education of caretakers, or combined home treatment of diarrhea and malaria.

To achieve maximum impact of zinc, we chose to provide zinc to caretakers in the home for several reasons. First, rural western Kenya did not have a well-established system of CHWs at the time of the study, as in other settings in Africa or south Asia. [7], [8], [20] Second, zinc was not yet available in public or private facilities and was virtually unknown in the community as a treatment for diarrhea. In such a context, we felt that to design a study where zinc was provided to local drug sellers and shops and expect mothers to purchase this new and unknown drug would not result in high levels of zinc usage, even at a minimal price. Third, zinc, along with ORS, is a good candidate for home treatment as it is inexpensive, easy-to-use, and has a low adverse event profile. [5] Mothers already commonly treat children’s illnesses at home on their own volition in many parts of Africa. [13], [20], [26] Although the design we used to deliver zinc and ORS at home is likely not scalable, with the advent of community-based health approaches like in Kenya, alternative strategies, including cost-effectiveness analyses, of delivery of home zinc and ORS to treat diarrhea need to be explored.

Although our study shows safe and proper use of zinc by caretakers at home, alternate approaches to increasing zinc use outside the clinic are possible. Treatment with zinc could be undertaken by community health workers, as in other places. [7], [8], [10] Moreover, zinc could be made more accessible to caretakers if it was available through non-facility sources of drugs, which in rural Africa could include CHWs through the iCCM strategy [29], [30] or drug sellers through local shops and pharmacies, as has been done for effective antimalarial treatment in many parts of Africa. [31].

Our study had several other potential limitations. First, our study was not designed to evaluate the impact of zinc the duration or severity of diarrhea episodes. Because zinc use has been shown to reduce diarrhea duration in multiple settings in several meta-analyses, we expected that in our setting, where approximately one-third of children were zinc-deficient, zinc would be effective in reducing diarrhea duration and did not need to be studied. [1], [5], [6] Second, zinc was only available for treatment in Lwak Hospital and not other area clinics during most of the study period. Therefore, zinc use was lower than in a setting where all clinics have zinc available. Nonetheless, if we doubled the percentage of diarrhea episodes treated with zinc to account for health-seeking at other area clinics, zinc use would still have been substantially lower (10.8%) among children receiving zinc in the clinics only compared with the children receiving zinc at home (61.2%). [19] Third, because we did not match or stratify villages on incidence of diarrhea or health-seeking at the time of village randomization, there were some baseline differences between village groups. Although we did adjust for baseline rates in our analysis of disease incidence, it is possible that residual confounding due to baseline differences by village group remained. Moreover, we did not have baseline rates of antimicrobial use, so could not adjust for baseline differences in the analysis of the impact of zinc on antimicrobial use, including antimalarials. Fourth, our findings on zinc’s impact on prevention might have been biased towards the null by including children younger than six months of age. There is no evidence that zinc use among children <6 months of age shortens duration of diarrhea episodes or prevents subsequent episodes [5], [32]–[34].

Since we undertook this study, the Kenya MOH has fully adopted zinc as part of the essential drug kit available in health facilities. Although we did not show a decrease in community incidence of diarrhea when zinc was available in the home, we did show that far more children with diarrhea in rural Kenya, and perhaps most areas of rural Africa, would likely be treated with zinc for diarrhea if zinc were either available in the home or at the community level, rather than in only health facilities. Adequate education, training and monitoring will be essential in adopting such a strategy. Assuming zinc has the same treatment and preventive impact in rural Africa as elsewhere, greater zinc use would translate into a beneficial public health impact.

## Supporting Information

Protocol S1 **Trial Protocol.**
(DOC)

Checklist S1 **CONSORT Checklist.**
(DOCX)
