# Supplementary material for: Village-Randomized Clinical Trial of Home Distribution of Zinc for Treatment of Childhood Diarrhea in Rural Western Kenya
Source: PLoS One. 2014 May 16;9(5):e94436. doi: 10.1371/journal.pone.0094436 (PMC4023937; doi:10.1371/journal.pone.0094436)
Supplement: Protocol S1 — Trial Protocol. (DOC) [file pone.0094436.s001.doc]

Clinical Effectiveness and Preventive Impact of Home Zinc Treatment for Acute Diarrhea in Children: A Cluster-randomized Field Trial in Rural Western Kenya

KEMRI SSC # 985; CDC IRB # 4678

Amended August 27, 2007

Investigators.

Daniel Feikin, MD, MSPH, Epidemiology Section Chief, CDC International Emerging Infections Program, Kenya.

Charles P. Larson, MD, SUZY Project Principal Investigator, Director, Health Systems & Infectious Diseases Division, International Center for Diarrheal Disease Research (ICDDR,B), Bangladesh

Robert F. Breiman, MD, Director, CDC International Emerging Diseases Program Kenya.

John Okanda, BSc. Nutritionist, CDC/KEMRI.

Mary J. Hamel, MD. Malaria Branch Chief, CDC/KEMRI Field Station, CDC

Laurence Slutsker, MD. CDC/KEMRI Field Station Director, Kisumu Kenya

Ana Wamae, MD. Director, Division of Child Health, Kenya Ministry of Health.

John Vulule, PhD. Director, Centre for Vector Biology and Control Research, Kenya Medical Research Institute

Kayla Laserson, PhD, Director, CDC/KEMRI Field station, Kisumu, Kenya

Abstract.

Background. Zinc deficiency is common in Africa. It has been shown in Asia that zinc as treatment for diarrhea can shorten the course of episodes of diarrhea, as well as prevent future episodes. The use of zinc at home to treat diarrhea in an African setting, where malaria, HIV and malnutrition are common, has not been well-studied.

Objective. To evaluate if zinc treatment for diarrhea given at home in Kenyan children will decrease the community prevalence of diarrhea more than zinc given only in the clinic

Work planned. We propose to do a community-randomized intervention study of 10 days of dispersible zinc tablets given in the home, in addition to ORS, to treat diarrhea in children under-5 years of age living in a rural part of Bondo District. The comparison group will be children who receive zinc and ORS in the clinic only. The primary outcome will be a comparison of the prevalence of diarrhea in home zinc versus nonhome zinc villages. Secondary outcomes will be the incidence of repeat episodes of diarrhea, the duration of diarrheal illness, the prevalence of acute respiratory infection, and the effect of malaria infection on treatment with zinc. Thirty-three villages (approximately 1300 children) will be enrolled and children will be followed for 1 year.

Significance of results. If this study shows zinc given at home to be effective, this might be considered by the Kenyan MOH as an essential component of the treatment of diarrhea in children at the community level.

Background.

Zinc deficiency has been found to be widespread among children in developing countries, and occurs in most of Latin America, Africa, the Middle East and South Asia (Shrimpton R 1993). Clinical and field studies have consistently observed an association between zinc deficiency and infectious disease morbidity (Srinivas U et al. 1988, Bahl R et al. 1998). Zinc deficiency is associated with a higher rates of infectious diseases, including skin infections, diarrhea, respiratory infections, malaria, and delayed wound healing (Aggett PJ & Comerford 1995, Prasad AS 1985, Bahl R et al. 1998). The higher rate of infectious diseases in zinc deficient children, however, is often multifactorial as they are often also malnourished and might have a preponderance of other risk factors for infection. Despite this caveat, some have estimated that based upon the impact of zinc deficiency on diarrheal disease, it is estimated zinc could prevent nearly 500,000 diarrhea related under-five deaths annually (Black RE et al. 2003, Jones et al. 2003).

Several recent randomized controlled trials have demonstrated the beneficial role of zinc in the prevention and treatment of diarrhea and pneumonia (Sazawal S et al. 1995 and 1998, Zinc Investigators Collaborative Group 1999, Zinc Investigators Collaborative Group 2000, Shanker AJ 2000, Fontaine O 2001, Bhutta ZA et al 2001, Baqui AH et al 2002, Strand TA et al. 2002, Bhandari H et al. 2002). Meta-analyses of published zinc treatment trials estimate that children between 3 months and 5 years of age who receive zinc for the treatment of a diarrheal illness (20 mg/day for 10 days) recover faster, with a 25% reduction in duration of illness and a 35% reduction in the severity of the illness. Furthermore, there is about a 30% reduction in the likelihood of developing prolonged diarrhea. Moreover, it has been shown that there is a beneficial effect of zinc treatment for children with and without zinc deficiency, although the effect is slightly greater in those with zinc deficiency (Zinc Investigators Collaborative Group 2000). These are the clinical treatment benefits. There are also longer-term beneficial effects. Over the 3 to 6 months following supplementation initiation, there is an estimated 30% decreased likelihood of a subsequent episode and 50% reduction in non-injury mortality (Zinc Investigators Collaborative Group 1999, Zinc Investigators Collaborative Group 2000, Fontaine O 2001, Bhutta ZA et al 2001, Baqui AH et al 2002, Strand TA et al. 2002). There are also preventive effects from use of zinc for treatment of diarrhea. In one community-randomized cluster study in Bangladesh, children in villages that received a 14 day course of zinc for diarrhea (completed 7 days on average), there was a 24% decrease in the duration of the diarrhea episode and a significant 15% decrease in diarrhea incidence over the study period (Bacqui AH et al 2002). There was also a 7% decrease in the incidence acute respiratory infections in the zinc villages compared with the control villages during the study period. Based on these data WHO and UNICEF came out with joint guidelines in 2004 for the inclusion of zinc in the treatment of acute diarrhea episodes for children under 5 years of age. (WHO/UNICEF Joint Statement 2004).

Why does zinc have a therapeutic and preventive impact on diarrheal illnesses? The available evidence of zinc’s potential role is derived from randomized clinical trials of zinc treatment or supplementation, that were not designed to explain why zinc is as effective as it seems to be. Zinc has been identified to play critical roles in function of metallo-enzymes, poly-ribosomes, cell membrane, and other cellular components, leading to the understanding that it also plays a central role in cellular growth and in function of the immune system (Shankar AH et al. 1998, Ibs K-H et al. 2003). With moderate zinc deficiency, epithelial barriers are compromised and multiple components of the immune system malfunction.

Although the zinc plasma pool is small, a concentration of 12-16 umol/L is extremely important for immune function in normally nourished children, it. Zinc deficiency in children is known to adversely affect innate and active immunity. Natural killer cell function, as well as phagocytosis by macrophages and neutrophils, are impaired by even mild zinc deficiency (Hasegawa W et al. 2000, Ravaglia G et al. 2000, Hock H et al. 2003, Shibuya A 2003). Also affected is the proliferation and function of cytotoxic T cells (Shankar AH et al. 1998). The obvious conclusion is that zinc deficiency results in diminished immunological competence, that in turn leads to an increased risk for infectious diseases and greater severity of illnesses.

At the urging of WHO and with their financial support, a French firm specializing in nutrition products for humanitarian aid (Nutriset, Ltd) and a French pharmaceutical laboratory (Rodael) developed a dispersible zinc tablet formulation. The zinc is encapsulated in a vanilla flavored coating that effectively masks the taste of zinc. Acceptability studies in Bangladesh, Tanzania, and elsewhereuniformly find the product is well received by young children (Nasrin D et al, in press). The dispersible tablets are packaged in a PVC/Alu blister pack of 10 tablets at an approximate cost of 14 cents US/blister pack. The current shelf life is 3 years and is expected to rise. It is stable in climates with high humidity and temperatures.

Although zinc use for the treatment of diarrhea has been well-studied in Asia, few studies of its use have been done in subSaharan Africa (Table 1). All three studies that evaluated the impact on diarrhea showed a decrease in diarrhea, although in one study this beneficial effect was only observed in growth-stunted children (Umeta M, 2002, Muller O, 2001, Makonnen B, 2003). However, in all of these studies zinc was given for prevention only, not for treatment. Two of these 3 studies also showed a decrease in ARI and fever. Two additional studies among African children evaluated malaria as the primary outcome – one from the Gambia showed a 32% decline in clinical malaria with zinc prophylaxis; however, a large multi-center study showed no change in parasitologically confirmed malaria . A large study of zinc supplementation for children in Pemba, Tanzania was recently published, showing a nonsignificant decrease in mortality among children who got zinc supplementation, although there was a significant decrease in those one year of age and older. (Sazawal S, et al 2007).

Table 1. Summary of studies of zinc on morbidity in Africa. Several more studies on growth parameters not included.

| Syndrome/site | How zinc given | Design | participants | 1^o^ outcome measured | How 2^o^ outcome defined | Results | Ref |
| --- | --- | --- | --- | --- | --- | --- | --- |
| Diarrhea |  |  |  |  |  |  |  |
| Ethiopia | 6 days/wk x 6 months | RCT, 50 zn, 50 placebo in both stunted and nonstunted | Stunted and nonstunted infants | Growth | History >3 loose stools/24 hrs | Significant decrease in stunted, not nonstunted | Umeta M, 2000 |
| Burkina Faso | 6 days/wk x 6 months | RCT, 709 enrolled | Children from a DSS | Malaria | “diarrhea” | Relative risk 0.87 (95% CI .79-.95) | Muller O, 2001. |
| Lesotho | 90 days daily after admission | RCT, 150 zn, 150 placebo | Children admitted with malnutrition | Multiple | History of diarrhea | Decreased in zn group at 30, 60, 90 days | Makonnen 2003 |
| ARI |  |  |  |  |  |  |  |
| Ethiopia | 6 days/wk x 6 months | RCT | Stunted and nonstunted | Growth | Cough | Significant decrease in stunted, not nonstunted | Umeta, 2000 |
| Burkina Faso | 6 days/wk x 6 months | RCT | From DSS | Malaria | Cough | No Difference | Muller, 2001. |
| Lesotho | 90 days daily after admission | RCT | Children admitted with malnutrition | Multiple | ? | Decreased in zn group at 30, 60, 90 days | Makonnen,, 2003 |
| Fever |  |  |  |  |  |  |  |
| Ethiopia | 6 days/wk x 6 months | RCT | Stunted and nonstunted | Growth | fever | Significant decrease in stunted, not nonstunted | Umeta, lancet 2000 |
| Burkina Faso | 6 days/wk x 6 months | RCT | From DSS | Malaria | fever | No Difference | Muller, 2001. |
| Lesotho | 90 days daily after admission | RCT | Children admitted with malnutrition | Multiple | fever | Decreased in zn group at 30, 60, 90 days | Makonnen, 2003 |
| Malaria |  |  |  |  |  |  |  |
| Burkina Faso | 6 days/wk x 6 months | RCT | From DSS | Malaria | Various ways | No Difference in incidence or severity | Muller O, 2001. |
| Uganda, Ghana, Tanz, Zambia, Ecuador | Supplement to treatment x 4 days | RCT, n = 1087 | Clinics | Malaria | fever, parasitemia, Hb | No difference | Zinc against Plasmodium group 2002. |
| Gambia | Twice weekly | ?, N=110 | Clinics | Malaria | clinical malaria | 32% reduction | Bates 1993. |

Other treatments, such as antimalarials, have been employed successfully using home-based treatment in Africa (Dunyo SK et al). There have been no studies of home zinc treatment for diarrhea in Africa. However, in Mali a program of community based zinc treatment of diarrhea by community health workers showed high acceptance and compliance by mothers (Ellis AA et al, 2006. Winch PJ et al, 2006). The major problem identified in the Mali project was that often mothers opted to give their children zinc alone when they had diarrhea accompanied by other symptoms, such as fever and cough, which required antimicrobial agents. (personal communication Peter Winch from the nonpublished manuscripted entitled “Lessons learned in a pilot introduction of zinc treatment for childhood diarrhea in Bougoni District, Mali”). This problem was addressed and rectified by re-education of community workers and mothers. This point will need to be emphasized and monitored closely in any program of home-based zinc treatment of diarrhea.

Important questions remain regarding the use of zinc in subSaharan Africa.

1. Will zinc used as an adjuvant treatment for diarrhea have the beneficial effects seen in Asian studies? The spectrum of pathogens causing diarrhea are different in many African settings. Whereas enterotoxigenic *E. coli* and cholera are common in south Asia, these pathogens are relatively rare as causes of nonepidemic diarrhea in Africa, where *Shigella*, *Salmonella*, *Campylobacter* and rotavirus might play more significant roles (Shapiro RL, et al, 2001, Brooks JT et al, 2003) . Could this difference in etiology of diarrhea lead to differences in the effectiveness of zinc?

2. 2. Will zinc be as effective in an area with high HIV prevalence? HIV infection could influence the spectrum of pathogens or the immunologic response to zinc treatment.

3. Lastly, does treating acute diarrhea with zinc also prevent future episodes of diarrhea? This could have important programmatic implications as introducing zinc as a treatment for diarrhea is likely to be easier than introducing it as a preventive medication because people tend to be more compliant with medicines viewed curative rather than preventive.

Rationale for amendment – August 2006: The Kenya Ministry of Health (MOH) has recently decided that it will institute the use of zinc supplementation for 10-14 days, along with low osmolarity oral rehydration solution, for the management of diarrhoea in children under 5 years of age presenting in health facilities. This is the recommendation put forward in the joint statement for the clinical management of diarrhoea by WHO and UNICEF (Joint Statement on the Clinical Management of Diarrhoea. Geneva – New York, World Health Organization-UNICEF, 2004. Document (WHO/FCH/CAH04.7) The new management guidelines are planned to be implemented in last quarter 2006.

Because of these new treatment guidelines, the previous study design outlined in this protocol, whereby some children do not receive zinc for diarrhoea treatment, is no longer ethical. Therefore, we are changing the study design to answer important supplemental questions related to the new guidelines. In particular, we will evaluate whether access to home treatment with zinc and ORS can have an additional impact on diarrhea reduction in addition to their use in health facilities. The Kenyan MOH is interested in the results of this study to decide whether to roll out zinc treatment to the community level.

Update on Amendment March 26, 2007: The procurement of zinc has been delayed by the Kenya MOH because of lack of competitive bidders in the tender sent out for this in late 2006. Now it is likely zinc will not arrive in Kenya until late 2007. Until zinc arrives and becomes part of the essential drug kit, the study will provide zinc treatment in the clinic to children coming in with diarrhea. The indications and dosages will those recommended by WHO and the same as will be adopted by the Kenya MOH. Until zinc becomes part of the essential drug kit and available to all children in the clinic, only children who are enrolled in this study will receive zinc. When zinc does become part of the essential drug kit and available in the clinics, the children in the study will continue to receive zinc as before, and nonstudy children will also receive it.

Justification for the study. The use of zinc to treat or prevent diarrhea in Kenya has not been studied. The results of such a study among Kenyan children will be important in providing relevant information to the Ministry of Health to make policy decisions and in building within-country advocacy for scale-up of zinc use and whether to adopt a policy of community use of zinc, in addition to use in the health facilities, for diarrhea treatment. Because access to health care is poor in much of Kenya and most children with diarrhea do not go to health facilities, home-based treatment of diarrhea with zinc could be reach more children, and treat and prevent more diarrhea.

Statement of the null hypothesis. In villages in Western Kenya where children with acute diarrhea are treated (self-prescribed zinc/provided in home) with zinc and ORS at home in addition to the clinic, the prevalence of severe acute diarrhea will be the same as in villages where children with acute diarrhea are treated with zinc and ORS at the clinic only.

Objectives

*Primary*

1. To assess if access to zinc treatment for diarrhea in the home in addition to zinc treatment of diarrhea in the clinic leads to a greater reduction in the prevalence of diarrhea than giving zinc for treatment of diarrhea in the clinic only

## Secondary

1. To assess if access to zinc treatment for diarrhea in the home in addition to zinc treatment of diarrhea in the clinic decreases the likelihood of recurrent diarrhea episodes
2. To assess if access to zinc treatment for diarrhea in the home in addition to zinc treatment of diarrhea in the clinic leads to a reduction in the prevalence of acute respiratory infections
3. To assess if access to zinc treatment for diarrhea in the home in addition to zinc treatment of diarrhea in the clinic results in reduction in antimicrobial use
4. To assess whether treatment of diarrhea with zinc in the clinic leads to a decrease in diarrhea prevalence from baseline rates of diarrhea before introduction of zinc
5. To assess difference in zinc utilization practices in parent initiated home treatment vs clinic treatment communities
   1. frequency of use and adherence to treatment instructions
   2. who receives zinc
   3. used for what indications
6. To compare cost estimates and estimate cost benefits
7. To assess acceptability and satisfaction with a home treatment program among providers and caretakers

# Study design

*Study site and population* -- This study will take place the area of Asembo within 4.25 kilometers of Lwak Hospital where CDC’s IEIP program has ongoing population-based surveillance for infectious disease syndromes.

Since September 2001, KEMRI and CDC have collaborated to operate a demographic surveillance system (DSS) in Bondo and Siaya Districts in Western Kenya. Two rural study sites near Lake Victoria, Asembo and Gem, are covered by the DSS, totaling a population of approximately 130,000 people living in approximately 34,000 households. The area is large (almost 400 km^2^), culturally homogeneous (95% Luo tribe); subsistence farming and fishing constitute the principal economy. The area has perennial, high-level malaria transmission and a high rate of HIV infection (2003, >10% men, >20% women aged 13-34 years). Other infectious diseases are also common in the area. Consequently, the area has mortality figures that reflect this burden of infectious diseases --- infant mortality rate (120 per 1,000 live births) and a life expectancy at birth of 38 years. The area is one of the most impoverished in Kenya; 60-70% of people live below the poverty line in Siaya and >70% in Bondo (Kenyan Central Bureau of Statistics, 1997). The area has high rates of migration -- in 2002, the rate of out-migration was 115 per 1,000 person-years and in-migration was 124 per 1,000 person-years.

The Lwak surveillance area includes 33 villages, with an approximate enrolled population of 23,000 people. Enrollment of villages began in October 2005 and was completed in June 2006. Homes are visited every two weeks by field workers who enquire about symptoms of key infectious diseases, like diarrhea, respiratory infections and malaria. For key symptoms, like diarrhea, cough and fever, the actual number of days of symptoms are recorded. Some basic measurements, such as temperature and respiratory rate, are taken. Health seeking in the last two weeks is also asked about. This is being done for children and adults.

*Inclusion criteria.* All children 2 to 59 months of age in households within selected villages that are already enrolled in the IEIP morbidity surveillance project (KEMRI SSC # 985; CDC IRB # 4678) will be eligible for enrollment. Children born into the study area after the start of the study will also be eligible for enrollment after they reach 2 months of age. Recruitment and enrollment will be done by field workers working for the study, who will read the consent form to guardians of all eligible participants that explains the study.

*Exclusion criteria.* Children under 2 months of age will not be eligible for enrollment, until they reach 2 months of age as the role of zinc has not been well-studied in neonates. Children of parents who do not give written informed consent for their participation will not be enrolled.

*Randomization.* This will be a cluster-randomized study. Thirty-three villages in Asembo participating in the morbidity surveillance (see below) will be selected to participate in this study. The villages will be randomized as 16-17 villages for zinc + ORS at home and clinic villages and 16-17 villages and ORS at clinic only villages. All eligible and participating children within a village will get the same treatment. A form will be filled out to keep track of the enrollment (appendix K). See figure.

Select 33 villages in Asembo closest to Lwak clinic

Obtain community approval

of chiefs and subchiefs

Home Zinc 10 days + low osm ORS

Referral to clinic

15 villages

No home zinc or ORS

Referral to clinic

15 villages

Continue biweekly household

visits for incident and prevalent

diarrhea, ARI, fever illness

Incident diarrhea episodes

Obtain parental consent of

children < 5 years

Baseline survey of prevalence

of diarrhea, ARI, malaria

Randomly assign to home zinc

and no home zinc villages

biweekly household

visits for incident and prevalent

diarrhea, ARI, fever illness

*Baseline survey and measurements.* All enrolled households will have a baseline survey done at the beginning of the study. The survey will collect the following data (Appendix B).

- Diarrhea management and care seeking practices
- Household characteristics
- Socio-demographic characteristics of household
- Water & sanitation exposures/practices
- Knowledge & attitudes about zinc, other micronutrients
- Height & weight
- Insecticide treated bednet use
- ORS awareness/use
- Antimicrobial use

### In addition, 165 children (5 per village selected randomly) will have a baseline serum zinc level measured, following parental informed consent (appendix J). This is to compare the baseline zinc status in the two groups. These same 165 children will also have a hemoglobin tested at the beginning of the study. Hemoglobin will be tested using a Hemocue machine. Children with anemia will be treated according to KMOH guidelines as follows:

### Hb 5-11 gm/dl - iron tablet or syrup and folate tablet for 14 days dosed according to age (Hb < 5 gm/dl – referral to hospital.

*Procedures for household visits.*  Field workers will visit each enrolled child every 2 weeks. S/he will ask about episodes of diarrhea since the last visit as well as current episodes. S/he will also record symptoms of acute respiratory illness and malaria, as well as any health seeking and medications resulting from these illnesses (Appendix C). S/he will ask about recent or current fever and any health seeking and medications resulting from the recent or current fever. S/he will also inquire about any use of zinc in the last two weeks. The number of days of zinc and number of pills of zinc will be documented. The interviewer will ask to see the zinc blister pack and record how many pills are remaining.

*Administration of zinc*

In the villages/clusters randomized to no home zinc, parents will be instructed by the field workers to take their children to the clinic if they have diarrhea, defined as an episode having 3 or more loose or watery stools (+/-) blood in a 24 hour period. At the clinic, the child will be evaluated by a clinical officer. If the child meets the above definition of diarrhea, they will be given zinc and ORS as described below.

In the villages/clusters randomized to home zinc, parents will be supplied with one 10-day blister pack of zinc and 3 satchets of ORS for each child in the house under 5 years of age. Zinc packs and ORS satchets will be given once every 2 months, regardless of whether the zinc was used in the previous month. Parents will be given instructions on how to use zinc and ORS at the beginning of the study and every 2 months thereafter when the zinc is given to them by a village reporter (a traditional birth attendant) or a community health worker. Parents in these villages/clusters will also be instructed to take their children to the clinic if they have diarrhea. They will be specifically instructed that taking zinc and ORS should not prevent them from taking their child to the clinic. Particular emphasis will be made that any child with diarrhea, *and* another symptom such as fever or cough, should definitely be taken to the clinic – that zinc is not adequate treatment for a child with fever or cough, who might need an antimalarial or an antibiotic. If the field worker on his biweekly visit finds out that the mother has given zinc to a child not meeting the diarrhea case definition, he will not tell the mother to stop the course of zinc. This is for several reasons. First, the field worker will not be medically trained and so will not be trained to apply the case definition of diarrhea. Second, diarrhea is a symptom taken by history, so that if the mother reports the child has diarrhea, the field worker will not really be able to assess the accuracy of that symptom. Third, there will be no adverse effects from the course of zinc – daily zinc supplementation regardless of illness has been shown to be beneficial in some studies (Zinc Investigators Collaborative Group 1999, Zinc Investigators Collaborative Group 2000). However, the field worker will encourage all mothers of children with diarrhea, especially those with fever, cough or another symptom, to go to the clinic to be seen by a clinical officer. The use of zinc for episodes of diarrhea will be evaluated on a biweekly basis based on the data collected by the field workers to assure that zinc is not being given alone when the child has other symptoms, such as fever and cough. If a significant number of mothers are giving zinc inappropriately without seeking care for other symptoms, further education in the use of zinc in the home will be given. Community health workers or village reporters, different from the field workers who do the biweekly visits, will make home visits every month during the first 3 months of the study to ensure that mothers understand the proper use of zinc and ORS for treatment of diarrhea. After the first three months, the village reporter or community health worker will visit the home every 3 months. On each home visit, the village reporter will ask some questions to assess whether mother is using zinc correctly (appendix L).

At the clinic, the child will be evaluated by a clinical officer. If the child meets the above definition of diarrhea, the clinical officer will inquire if they are already taking zinc for this episode of diarrhea. If they are not already taking zinc and ORS, he will give them zinc and ORS as described below.

Along with instructions regarding how to take zinc, parents will be instructed in the use of ORS and proper fluid management (i.e. encouragement of breastmilk, if breast-feeding, ORS, or food-based fluids, such as soup, enriched uji). Recommended ORS amounts will follow KMOH guidelines. In the first 4 hours, the amount of ORS recommended will be as follows:

| Age | Up to 4 mos | 4-11 mos | 12 mo – 2 yr | 2-5 yrs |
| --- | --- | --- | --- | --- |
| Weight | < 6 kg | 6-<10 kg | 10-<12 kg | 12-19 kg |
| In ml | 200-400 | 400-700 | 700-900 | 900-1400 |

This should be followed by replacement for each loose stool as follows: up to 2 years of age 50-100 ml per stool and 2 years or more 100-200 ml.

Zinc will be administered using the same formulation in the home and in the clinic. Zinc will be administered in the form of a dispersible tablet of zinc-sulfate, 20 mg. A half-tablet, 10 mg, will be administered to children between 2 and 5 months of age. A full 20 mg tablet will be given to children > 6 months of age. The tablet will be dissolved in water, ORS or breast milk and administered to the child. . Mothers will be instructed on using clean water to dissolve tablets for remaining doses. Children who vomit after taking their dose of zinc will not be given another dose that day. The mother will be instructed how to administer the zinc tablet. Zinc will be given once daily for 10 days total. Zinc will be provided in a dispersible zinc tablet and packaged in a 10 tablet blister pack. If the child is holding down fluids and has not vomited in the past 2 hours, then zinc treatment should be started, otherwise the caretaker will be instructed to wait until this occurs.

*Referrals.* If on the day of the field interviewer’s visit, the child has any of the following conditions on the day that the field worker visits the home, the child will be referred to the health clinic

- Diarrhea with
  - moderate to severe dehydration (defined according to IMCI as having 2 or more of the following symptoms: lethargic/unconscious, sunken eyes, not able to drink or breastfeed, skin pinch goes back slowly, restless/irritable) or not able to take ORS
  - Continued illness by the 7^th^ day observation
  - bloody diarrhea
  - persistent diarrhea at any point in the study will be referred to Lwak hospital . Persistent diarrhea is defined as > 14 days of diarrhea.

### History of fever in the last 24 hours

- Cough and difficult or fast breathing

Care at Lwak clinic for children in the study will be free of charge. Children in the study will be identified at the clinic by an identification card.

*Clinic visits.* All study children will be identified in the clinic by an identification card. They will be seen by a clinical officer hired by the study. Diagnosis and treatment will be according to standard protocols used for childhood illness in peripheral health facilities in Kenya (IMCI. Zinc and low osmolarity ORS will be administered according to MOH protocol. For children in the home zinc group, the clinical officer will inquire if the child is already taking zinc for that episode of diarrhea. If so, then no more zinc will be given. If not, then a 10 day course of zinc will be given. Bloody diarrhea will be treated with nalidixic acid. Uncomplicated malaria will be treated according to KMOH guidelines, artemether/lumefantrine (coartem) as of June 2006. Pneumonia not requiring referral to hospital will be treated with cotrimoxazole or amoxacillin. Children meeting criteria for referral to the hospital according to IMCI guidelines will be hospitalized at Lwak Hospital, and transferred to Bondo District Hospital or Nyanza Provincial Hospital, if appropriate treatment cannot be given at Lwak..

Children with a recent history of or documented fever will have a blood smear for malaria taken. If the results of the blood smear are positive for malaria, the child will be treated for malaria as outlined above. Blood smears will be read by a microscopist at the clinic.

A questionnaire will be filled out for study children who visit the clinic. The questionnaire will ask about symptoms, duration of illness and health-seeking behaviour (Appendix E).

*Follow-up of cases of diarrhea.* Children identified as having a new episode of diarrhea on the routine biweekly household visit or in the clinic will be visited 7 days later. Referrals will be made on these visits according the criteria listed above under G. A short questionnaire regarding ongoing symptoms and signs of illness, interim health care seeking, and possible side effects to the study medication will be administered (Appendix D). No additional zinc or ORS will be given by the field worker on follow-up visits. If the child is still having significant diarrhea at the follow-up visits, the field worker will instruct the mother to take the child back to the clinic (or to the clinic if they have not gone yet.) The child will next be visited on day 14 according to the regular weekly visit schedule.

*Definition of study outcomes.*

- 1. Diarrhea – A diarrheal day will be considered a day in which the child has 3 or more loose or watery stools. New episodes of diarrhea will be defined as at least one diarrheal day without diarrhea in the previous 72 hours. A severe diarrheal episode will be defined as an episode in which children go to the clinic to receive care for the episode.
  2. Acute Respiratory Infections.
     1. Clinical ALRI - An episode of acute lower respiratory illness (ALRI) will be defined for the home visits as a history of cough or difficulty breathing for > 2 days. An ALRI day will be considered a day that is part of an ALRI episode in which a child has cough or difficulty breathing. New episodes of ALRI will be defined as an ALRI episode without an ALRI day in the previous 72 hours. Acute respiratory infection (ARI) will include any child with cough (of any duration), difficulty breathing, or ear pain and/or discharge.
     2. Pneumonia – Pneumonia and severe pneumonia will be defined for children who visit the clinic and meet the IMCI criteria:
        1. Pneumonia in children < 5 years of age -- Cough or difficulty breathing *and* Respiratory rate > 50/minute for 2-11 month old, >40/minute for 1-4 years
        2. Severe pneumonia in children 1 month - 5 years of age

Cough or difficulty breathing and

- - - - 1. chest indrawing
        2. stridor in a calm child
        3. a general danger sign

Unable to breastfeed or drink

Vomits everything

Convulsions

Lethargy or unconscious

- 1. Malaria -
     1. Clinical malaria – History of fever in the preceding 24-48 hours as reported by the caretaker
     2. Parasitologic malaria – History of recent fever or documented fever and a blood smear positive for asexual malaria parasites.

*Adjuvant therapies*. Children who have not received Vitamin A in the prior 6 months in both groups will be given Vitamin A at the start, 6 months, and end of the study, according to KMOH guidelines, dosed according to age. Children in both groups 2 years of age or older who have not received mebendazole or albendazole in the prior 6 months will receive mebendazole 500 mg at the start, 6 months, and end of study, according to KMOH guidelines.

*Compliance monitoring*. Compliance will be monitored by asking the child’s caretaker how many pills she gave the child. In addition, caretakers will be asked to keep the blister pack and the number of pills remaining on the day 7 and 14 day visit will be counted and recorded.

## Adverse Events and Incidents

The recording and reporting of adverse events and incidents fall into two categories – solicited adverse events and reporting of incidents to Human Research Committees. These two categories will be described separately.

## Solicited Adverse Events.

Solicited Adverse Events are potential side effects to the treatment of diarrhea, zinc and/or ORS that occur up to 24 hours after the last dose, and will be ascertained from the 3 and 7-day visits by the field worker using a standardized questionnaire (Appendix E). These may or may not be reportable incidents, depending on whether they meet the definitions below.

### Definitions for solicited adverse events

Solicited adverse events include the systemic reactions of fever, irritability, nausea, vomiting, and rash. (Appendix D)

Definitions of Solicited AEs:

Fever: as reported by the mother/caretaker or the field worker taking axillary temperature of ≥38ºC (100.4ºF), recorded by digital thermometer. Severe defined as > 72 hours of fever after administration of zinc and/or ORS.

Irritability. Activity or behaviour deemed to be unusual in intensity or duration compared with child’s normal behaviour as reported by the mother/caretaker. Severe defined as > 72 hours of irritability after administration of zinc and/or ORS.

Nausea. Complaint of being nauseated in verbal children as reported by the mother/caretaker after administration of zinc and/or ORS.

Vomiting. An episode of vomiting will be defined as at least one episode of vomiting occurring within 60 minutes of zinc administration in a child previously stable and not having vomited for at least 60 minutes prior to receiving zinc. Severe defined as not being able to breastfeed or drink anything for > 72 hours after administration of zinc and/or ORS, as reported by the mother.

Rash. Unusual redness, texture or swelling of skin that was not present before administration of study drug. Severe defined as involving mucosal membranes at any time, or having a duration of > 72 hours after administration of zinc and/or ORS.

### Reporting of incidents

The latest CDC guidelines for reporting of incidents associated with the study will be followed. Reportable incidents fall into four categories.

- Unanticipated problems involving risks to subjects or others
  - Including serious, unexpected, and possibly related adverse events
  - Other unanticipated problems involving risks to subjects
- Breaches of protocol and other examples of noncompliance with protocol or IRB requirements
- Premature suspension or termination of study
- Other incidents specified in the protocol (none apply here) or requested by IRB

Not all adverse events will require an incident report. Adverse events requiring an incident report must be meet all 3 criteria of being serious, unexpected, and possibly related adverse events. Serious AEs in the context of this study result in death, are life-threatening, result in inpatient hospitalization, or cause permanent disability. Unexpected AEs refer those that have not been observed before with this intervention. Expected AEs from the intervention should be clearly defined in the consent form. For this study, vomiting after the first dose of zinc is an expected AE that has been observed before. However, protracted vomiting would be unexpected. A reportable AE must be at least possibly related to the study intervention, zinc. That is it must be at least as likely to have been due to zinc as to other causes.

The procedure for reporting incidents is as follows. An Incident Investigation Form (Appendix F) will be initiated by the field supervisor, clinical officer or study coordinator upon notification of a possible incident. It will be completed by the PI, who will determine if it is a reportable incident. In the case that the PI is not available (e.g. on leave, tdy, etc), a designated senior scientist at CDC/KEMRI will be asked to make this determination. If deemed a reportable incident, within 2 days of being notified of the incident, the incident should be reported informally by email to both CDC Human Research Program Office and KEMRI ERC with the protocol number, a statement that an incident has occurred, the site and nature of the incident. Within 2 weeks of being notified of the incident, the program staff, particularly the PI, should submit an incident form 0.1254 (Appendix G) to CDC HRPO and KEMRI ERC, which includes a description of the incident and actions taken. If the incident is a reportable adverse event, then an adverse event form (0.1254Sv1.0, Appendix H), which documents the type of adverse event, the outcome, the seriousness, and the relatedness to the study intervention.

After review of the documentation, CDC IRB will notify the PI through HRPO of any further actions to be taken. These actions could include requests for further information, changes to the protocol, suspension or termination of the study, or acceptance of the report and action already taken with no further recommended actions. KEMRI ERC will be informed of CDC IRB decisions and may provide addition recommendations for further actions, if necessary.

Identification of reportable incidents will be accomplished by study personnel. The PI and study coordinator will identify breaches of protocol. Reportable adverse events will come to the attention of the PI through the community interviewers during their follow-up visits after starting treatment for diarrhea or at any other time in their biweekly rounds. Also, the clinical officer in the clinics will report potential adverse events in children that he sees in the clinic.

# Data Management

*Data collection.* Data forms will initially be field tested for ease of use, ease of analysis, and proper collection of desired information. Questionnaires will be completed by trained household interviewers and health facility recorders. Responses will be written on a hard copy of the questionnaire in ink. After review in the field, hard copies of the questionnaires will be transferred by project vehicles to the site of data entry (see below). Forms should stay in the field no longer than 1 week after completion for review by the field supervisor.

*Data entry and storage.* Data will be collected on scannable forms. Forms will be scanned using a commercial software package (TELEforms Enterprise, Verity) at the CDC/KEMRI field station in Kisian. Scanned data will be reviewed by data specialists and corrected where appropriate using the TELEforms software. A pilot test of paperless data collection using pocket Personal Digital Assistants (PDAs) will be done and compared to the data collected on scannable forms. If this pilot shows that the PDAs are equivalent to scannable forms in data quality, PDAs will be adopted as the primary data collection system. Data from both scannable forms and PDAs will be stored in a database in Microsoft Access and maintained on the network in a restricted drive at Kisian. The database will be routinely backed up. Cleaning programs will be run each week to identify errors in the data collection. When applicable, the forms will be sent back to the field for correction of errors. After data entry, hard copies of forms will be stored in locked long-term storage units in Kisumu (CDC). Access to long-term storage will be restricted to relevant project staff.

*Linkage of household morbidity surveillance data to DSS data.* Data from this study at the Lwak site will be linkable to relevant DSS data (Protocol SSC #647, CDC #3308). Linkage will be based on the DSS ID number of the participating child. This information will be used to compare demographic characteristics of children in zinc and placebo villages, as well as children with diarrhea and without. Permission to link DSS data to data from this study will be requested in the informed consent form (see below).

*Linkage of laboratory results and morbidity surveillance data.* On-going surveillance for diarrheal disease among children will continue in Lwak clinic (protocol SSC#381 , CDC#2616). As part of this protocol, all persons presenting with diarrhea to one of 6 clinics in Asembo and Gem, including Lwak, who can produce stool while in the clinic, will have a stool culture sent to the lab at Kisian for microbiologic identification and antimicrobial susceptibility testing. These results will be able to be linked to the zinc study by ID number of the individual, which will be present on all forms. Permission to link microbiologic stool data to data from this study will be requested in the informed consent form (see below).

*Data access.* Data in Kisumu will be accessible to a limited number of relevant CDC/KEMRI and ICDDRB staff.

*Confidentiality of data.* Data will be kept as confidential as permitted by law. Hard copies of questionnaires will be stored at all times in a room that will be locked when not occupied. Access to these files will be limited to relevant persons. Electronic copies of the data will be kept in the above-mentioned databases, which will only be accessible to relevant CDC/KEMRI and ICDDRB staff and will be password protected.

Data Analysis

All statistical analyses will be performed using SAS system for Windows (SAS Institute, Cary, NC).

*Calculation and comparison of prevalence rates*

Prevalence rates of diarrheal disease will be calculated as number of diarrheal days per person-time. Using person-time as the denominator can incorporate information from children who are in the study for only part of the time, contributing less than 1 full year of data, due to out-migration, death or birth. Curves of monthly prevalence rates of diarrhea will be calculated and plotted for home zinc and nonhome zinc villages. 95% confidence intervals will be calculated around these point estimates of prevalence rates. Because of the study design in which zinc will be given only for the treatment of diarrhea, the preventive effects of zinc will likely become more apparent with time as more children receive zinc. Therefore, initially the prevalence will be the same. With time, the prevalence might diverge between the study groups. Prevalence rates over time between the home zinc and nonhome zinc groups will be compared using Poisson regression applying the generalizing estimating equation (GEE) technique to account for the repeated measures design and control for correlation of diarrheal days among individuals. The Wald test will be used to evaluate the parameter that estimates the effect of treatment group. Statistical significance will be considered at the 0.05 level. Potential confounders of the outcome, such as village of residence and age of the child , will be controlled for in the final model.

Prevalence rates for clinically suspected acute respiratory illness based on the caretakers reports every 2 weeks will be calculated in the same way as for diarrhea.

In addition, we will analyze the cumulative dose effect (total number of zinc days) on the prevalence of diarrhea among individuals in the zinc villages using poisson regression with total number of zinc days as the independent variable.

## Calculation and comparison of incidence rates

Incidence density rates of diarrheal disease will be calculated as number of new episodes of diarrhea per person-time. Using person-time as the denominator can incorporate information from children who are in the study for only part of the time, contributing less than 1 full year of data, due to out-migration, death or birth. 95% confidence intervals will be calculated around point estimates of incidence rates. Incidence rates for acute respiratory illness and malaria will be calculated in the same way as for diarrhea. The mean number of episodes of diarrhea will be compared between the home zinc and nonhome zinc groups using an ANOVA test accounting for repeat episodes of diarrhea within the same child, accounting for potential confounding variables, such as age and season. Potential confounders of the outcome, such as village of residence and age of the child and malaria coinfection, will be controlled for in the final model.

*Calculation and comparison of time to repeat episode of diarrhea.*

The time before a repeat episode of diarrhea in children who have their first episode of diarrhea will be compared between the home zinc and no home zinc groups. The time to a new episode of diarrhea will be measured as the number of days after the last day of the child’s first episode of diarrhea to the first day of the next episode of diarrhea in that child. This analysis will use as a Cox proportional hazards model, in which the child will be censored after the first day of their first repeat episode of diarrhea.

A secondary analysis will compare the number of repeat diarrheal episodes that occur after the first episode of diarrhea. Mean or median number of repeat episodes will be compared by t-test or Wilcoxon rank sum test depending on the distribution of episodes.

*Comparison of the effect of zinc on the treatment of diarrhea.* The mean duration of diarrheal episodes between children in the home zinc and nonhome zinc groups will be compared using ANOVA controlling for potential confounders such as age and season. Repeat episodes of diarrhea in the same children will be accounted for in the analysis. We will also analyze differences in the severity of diarrhea between groups (severe defined as visiting the clinic) and antibiotic use between groups. This will be done using logistic regression with clinic visit and use of antibiotic as the outcome variable.

## Sample size.

*Diarrhea Prevalence*.

The below calculations are for our primary study outcome of diarrhea prevalence.

Table 2a. Sample size estimates for various prevalences and reductions. Sample size table assumes a 5% reduction in diarrhea prevalence in the control group with a difference of 15% - 10% between groups.

| Difference in groups | Initial Prevalence in each group | N for each group initially/Total N | N for each group at end of trial/ Total N  (with 15% attrition) |
| --- | --- | --- | --- |
| 15% | 3.6 | 1059/2118 | 900/1800 |
| 14% |  | 1184/2368 | 1006/2012 |
| 13% |  | 1318/2636 | 1120/2240 |
| 12% |  | 1435/2870 | 1219/2438 |
| 11% |  | 1651/3302 | 1403/2806 |
| 10% |  | 3036/6072 | 2580/5160 |

Table 2b. Sample size estimates for various prevalences and reductions Sample size table assumes a 10% reduction in diarrhea prevalence in the control group with a difference of 15% - 10% between groups.

| Difference in groups | Initial Prevalence in each group | N for each group initially/Total N | N for each group at end of trial/ Total N  (with 15% attrition) |
| --- | --- | --- | --- |
| 15% | 3.6 | 1550/3100 | 1317/2634 |
| 14% |  | 1745/3490 | 1483/2966 |
| 13% |  | 1978/3956 | 1681/3362 |
| 12% |  | 2259/4518 | 1920/3840 |
| 11% |  | 2798/5596 | 2378/4756 |
| 10% |  | 4118/8236 | 3500/7000 |

We will assume 3.6 episodes per year of diarrhea in children in the study area, with an average duration of 3 days +/- 1 day. This gives a yearly prevalence of diarrhea of 10.8 days of diarrhea per child per year with a range of 7.2 to 14.4 days. We will also assume that because zinc use will increase gradually in the zinc villages as more children get diarrhea, it will take 6 months to achieve the indicated difference in diarrhea prevalence between the two groups (column 1). In the top table, it is assumed that there will be 5% reduction in diarrhea prevalence in the control group (nonhome zinc group) between the start of the study and end of the study 12 months later, due to the implementation of zinc in the clinic. The bottom table assumes that this reduction in the control group over the 12 months will be 10%.

In this repeated measures, longitudinal study design, correlation between events must be accounted for. That is, some children will be more likely to have repeated diarrheal episodes and so will contribute disproportionately to the community prevalence. To do this, sample size calculations accounted for correlation by using the generalized estimating equation (GEE) method described by Rochon (Rochon J, 1998).

Approximately 14% of enrolled participants in the morbidity surveillance study are under 5 years of age. Therefore, we would expect in a population of 25,000 enrolled participants in the 33 villages in Lwak approximately 3,000 children under 5 years. Therefore, we will have the power to detect a difference of 12% between home zinc and nonhome zinc groups, assuming a 5% decrease in diarrhea prevalence in the nonhome zinc group during the study period, or a 15% difference between the home zinc and nonhome zinc groups, assuming a 10% decrease in diarrhea prevalence in the nonhome zinc group over the study period.

# Time frame

*Training.* A two-week training will be conducted to train field workers in administration of consent, questionnaire administration, use of ORS and zinc administration and referral criteria.

*Consent phase.* All eligible households in the 33 selected villages will be offered participation in the surveillance. In each household with a child under 5 years of age, a caretaker of the child must sign an informed consent form allowing participation of the child. The consent phase will take approximately 2 weeks. Community consent will be obtained by having an initial meeting with the chiefs and subchiefs of the area, followed by biannual update meetings. In addition, barazas (talks) will be held with community groups, such as churches and women’s groups, to sensitize them to the project.

*Length of study.* The active phase of the study in which weekly household visits will be made will last 1 year.

*Expected time to publication.* The study will be analyzed and prepared to submit for publication 6 months after the end of the study.

Ethical considerations

*Risks.* Zinc has been administered to over 35,000 children in the ICDDR.B Cholera Hospital in Dhaka without excess mortality.

Zinc can lead to an increase in vomiting. In one study in Nepali children there was a two-fold increase in vomiting (Strand, 2002). Another recent study in Bangladesh showed that more children vomited in the course of their treatment when taking dispersible zinc tablet than among children who did not get zinc -- overall 20% vomited; 40% due to zinc, 26 % placebo, and 34% due to the illness. (Larson 2005). There was also an increase in regurgitation in children who got zinc versus those who did not (8.6% zinc 4.1% placebo, 1.5% nothing. However, it should be noted that most children who vomited after zinc administration had a history of vomiting within the last 24 hours. Moreover, 91% of children who vomited after zinc administration only vomited once.

A small bruise or mild pain on the finger from where the fingerstick blood sample is taken may develop.

The only other risk of being in the study will be minor inconveniences to the family resulting from the brief amount of time necessary to complete the questionnaires every other week, or on days 3 and 7 after diarrheal episodes.

*Benefits.* As mentioned, zinc has been shown in some studies to shorten the course of diarrhea, and to prevent recurrent episodes. ORS will be given to all children with diarrhea, which can prevent serious dehydration. Zinc and ORS will be given at home to children in villages in the home treatment arms – ORS will be given at home to the children in the villages without home treatment. In both arms, instructions will be given to parents to take children with severe diarrhea, fever, and cough or difficulty breathing to Lwakclinic, where they are entitled to free care as part of the IEIP surveillance study – this free care is available to both zinc and nonzinc households. In addition, a full stock of antibiotics and ORS will be maintained at the clinic by the study. Children with persistent diarrhea will be referred to the hospital. Children will receive deworming medication and vitamin A in both study groups.

*Informed consent.* We will ask the caretaker of the child to give informed consent for the child to participate in the study during the study’s 1 year duration. The voluntary nature of this study will be emphasized and they will be told that they can refuse at any time. Permission to link data from this study with data from the DSS (Protocol SSC #647, CDC #3308) and from the etiology of diarrhea surveillance study (SSC #381, CDC # 2616) will be sought. Individuals in the participating households will have an identification card made that identifies them as a participant in the surveillance project.

Each card will have a photo of the person to prevent lending of cards to nonstudy persons. This card will entitle the person to free medical care at the designated clinic. Informed consent forms will be translated into the vernacular language of the area, dho-Luo. It will be back translated into English before the translation is finalized.

Expected application of the results

Data on the effectiveness of home zinc treatment for diarrhea on the prevention of diarrhea (and ARI and malaria) in the community will be shared with the Kenyan Ministry of Health, Division of Child Health. It will assist the MOH in evaluating the potential impact of introduction of widespread home zinc use. These data will also be used by CDC-Kenya to formulate future research agendas. Lastly, the information obtained from this study will contribute to the body of literature on zinc use in Africa and might be of use to other African countries in their advocacy of introduction of zinc as a treatment for diarrhea.

# References

Aggett PJ & Comerford JG. Zinc and human health. *Nutrition Reviews* 1995 ; 53 : S16-

S22.

Bahl R, Bhandari N, Hambidge KM & Bhan MK. Plasma zinc as a predictor of diarrhea and respiratory morbidity in children in an urban slum setting. *American Journal of Clinical Nutrition* 1998 ; 68 : 414S-417S.

Bahl *et al*. Effect of zinc on clinical course of acute dairrheoa. J Health Popul Nutr Dec 2001 :339-346.

Baqui AH, Black RE, El Arifeen S, Yunus M, Chakraborty J, Ahmed S, Vaughan JP. Effect of zinc supplementation started during diarrhea on morbidity and mortality in Bangladeshi children: community randomised trial. BMJ 2002 Nov 9;325(7372):1059.

Bates CJ, Evans PH, Dardanne M, etal. A trial of zinc supplementation in young rural Gambian children. Br J Nutr 1993;69:243-55.

Bhandari N, Bahl R, Taneja S *et al*. Effect of routine zinc supplementation on pneumonia in children aged 6 months to 3 years: randomised controlled trial in an urban slum. BMJ. 2002 Jun 8;324(7350):1358.

Bhutta ZA, et al. Prevention of diarrhea and pneumonia by zinc supplementation in children in developing countries: pooled analysis of randomized controlled trials. Zinc

Investigators Collaborative Group. J Pediatr 1999;135:689-97.

Black RE . Therapeutic and preventive effects of zinc on serious childhood infectious diseases in developing countries. *American Journal of Clinical Nutrition* 1998 ; 68S : 476S-479S. International Centre for Diarrhea Disease Research, Dhaka, Bangladesh. Zinc supplementation in the treatment of childhood diarrhea. Indian J Pediatr 1995;62:181-93.

Black RE , Sazawal S. Zinc and childhood infectious disease morbidity and mortality. British Journal of Nutrition. 2001; 85 (suppl 2) :S125-S129.

Black RE, Morris SS, Bryce J. Where and why are 10 million children dying every year?

*Lancet* 2003; 361:2226-34.

Brooks JT, Shapiro RL, Kumar L, et al. Epidemiology of sporadic bloody diarrhea in Western Kenya. Am J Trop Med Hyg 2003;68:671-7.

Castillo-Duran C, Vial P, Uauy R. Trace mineral balance during acute diarrhea in infants. *J Pediatr* 1988; 113: 452-457.

Dunyo SK, Afari EA, Koram KA et al. Health centre versus home presumptive diagnosis of malaria in southern Ghana: implications for home-based care policy. Trans R Soc Trop Med Hyg 2000;94:285-8.

Ellis AA, Winch P, Doua Z, Gilroy KE, Swedberg E. Home management of childhood diaorrhea in southern Mali – Implications for introduction of zinc treatment. Soc Sci Med 2006. doi:10.1016/j.socscimed.2006.10.011.

Faruque ASG, Mahalanabis D, Haque SS, Fuchs GJ, Habte D. Double-blind, randomized, controlled trial of zinc or vitamin A supplementation in young children with acute diarrhea. Acta Paediatr 1999;88:154-60.

Fontaine O. Effect of zinc supplementation on clinical course of acute diarrhea. J Health Popul Nutr 2001; 19(4):339-346.

Hidayat A, Achadi A, Sunoto, Soedarmo SP. The effect of zinc supplementation in children under three years of age with acute diarrhea in Indonesia. Med J Indonesia 1998;7:237-41.

Holz C, Peerson JM, Brown KH. Suggested lower cutoffs of serum zinc concentrations for assessing zinc status: Reanalysis of the Second National Health and Nutrition Examination Survey data (1976-1980). Am J Clin Nutr 2003;78:756-64.

Ibs K-H, Rink L. Zinc altered immune function. J Nutr 2003;133(Suppl):1452S-1456S.

Jones G, Steketee RW, Black RE, Bhtta ZA, Morris SS, Bellagio Child Survival Study Group. How many child deaths can we prevent this year? *Lancet* 2003;362:65-71.

Larson CP, Hoque AB, Larson CP, Khan AM, Saha UR. Initiation of zinc treatment for acute childhood diarrhoea and risk for vomiting or regurgitation: a randomized, double-blind, placebo-controlled trial. J Health Popul Nutr 2005;23:311-19.

Makonnen B, Ventaer A, Joubert G, A randomized conrrolled study of the impact of dieteary zinc supplementation in the management of children with protein-energy malnutrition in Lesotho. I: Mortality and morbidity. J Trop Ped 2003;49:340-52.

Muller O, Becher H, Van Zweeden AB, et al. Effect of zinc supplementation on malaria and other causes of morbidity in west African children: randomized double blind placebo controlled trial. BMJ 2001;322:1-6.

Prasad AS. Clinical manifestations of zinc deficiency. *Annual Review of Nutrition* 1985 ; 5 : 341-363.

Rochon J. Application of GEE procedures for sample size calculations in repeated measures experiments. Statistics in Medicine 1998;17:1643-58.

Roy SK, Tomkins AM, Akramuzzaman SM, et al. Randomised controlled trial of zinc supplementation in malnourished Bangladeshi children with acute diarrhea. Arch Dis Child 1997;77:196-200.

Sachdev HPS, Mittal NK, Mittal SK, Yadav HS. A controlled trial on utility of oral zinc supplementation in acute dehydrating diarrhea in infants. J Pediatr Gastroenterol Nutr 1988;7:877-81.

Sazawal S, Black RE, Bhan MK, Ghandari N, Sinha A, Jalla S. Zinc supplementation in young children with acute diarrhea in India. N Engl J Med 1995;333:839-44.

Sazawal S, et al. Zinc supplementation reduces the incidence of acute lower respiratory

infections in infants and preschool children: a double-blind, controlled trial.

Pediatrics 1998;102:1-5.

Sazawal S, Black RE, Ramsan M, et al. Effect of zinc supplementation on mortality on children aged 1-48 months: a community based randomized placebo controlled trial. Lancet 2007:369:927-34.

Shankar AH & Prasad AS. Zinc and immune function: The biological basis of altered resistance to infection. *American Journal of Clinical Nutrition* 1998 ; 68 : 437S-463S.

Shanker AJ. Nutritional modulation of malaria morbidity and mortality. J Inf Dis 2000;182(Suppl 1):S37-53.

Shapiro RL, Kumar L, Phillips-Howard P, et al. Antimicriobial resistant bacterial diarrhea in rural Western Kenya. J Infect Dis 2001;183:1701-4.

Shrimpton R. Zinc deficiency- is it widespread but under-recognized? I: Subcommittee on Nutrition News. Vol 9, Geneva : United Nations Administrative Committee on

Coordination. 1993 .

Strand TA, Chandyo RK, Bahl R, Sharma PR, Adhikari RK, Bhandari N, et al.

Effectiveness and efficacy of zinc for the treatment of acute diarrhea in young

children. Pediatrics 2002 May; 109 (5):898-903.

Umeta M, West CE, Haidar J, Deurenberg P, Hautvast JG. Zinc supplemention and stunted infants in Ethiopia: a randomized controlled trial. Lancet 2000;3555:2021-6.

WHO/UNICEF Joint Statement on clinical management of acute diarrhea. May 2004. Accessed at http://whqlibdoc.who.int/hq/2004/WHO_FCH_CAH_04.7.pdf

Winch PJ, Gilroy KE, Doumbia S, et al. Short report: Prescription and administration of a 14 day regimen of zinc treatment and childhood diarrhea in Mali. Am J trop Med 2006;74: 880-3.

Zinc against Plasmodium Study group. Effect of zinc on the treatment of Plasmodium falciparum malaria in children: a randomized controlled trial. Am J Cin Nut2002;76:805-12.

Zinc Investigators’ Collaborative Group. Prevention of diarrhea and pneumonia by zinc

supplementation in children in developing countries : pooled analysis of

randomised controlled trials. J Pediatr 1999; 135: 689-97.7-7.

Zinc Investigators' Collaborative Group. Therapeutic effects of oral zinc in acute and persistent diarrhea in children in developing countries: pooled analysis of randomized controlled trials. *Am J Clin Nutr* 2000; 72: 1516-1522

Appendix A. Informed Consent for zinc effectiveness study – (Flesch-Kincaid readability score – Grade 6.9 )

| Today’s date | □□/□□/□□ | Village/Compound, House | □□□/□□□,□ |
| --- | --- | --- | --- |
| Name of compound head | |  | |

CDC KEMRI would like to do a new research study on diarrhea in the Asembo area. This study will look at whether a vitamin called zinc shortens the number of days of diarrhea in children. We also want to know if zinc protects against repeat diarrhea illnesses and pneumonia. We will be comparing zinc given for diarrhea in the clinic and in the home. Zinc is a vitamin that is in some foods, but can also be given as a pill. Some children with diarrhea in the study will get zinc at home and also the clinic and some will get it only in the clinic.

Your house has been picked because you have a child less than 5 years of age and you are enrolled in the IEIP study . You can decide if your child joins this study. It is your free choice.

What we would like to do: If you are in a village that will get zinc in the home, we will give you a 10 day course of zinc tablets every two months. If your child gets diarrhea, we ask that you give him or her the zinc tablets, one 20 mg tablet a day for 10 days, for children from 6 months to 5 years of age; and one-half tablet (10 mg) a day for 10 days for children from 2 months to 5 months of age. . We will also give you oral rehydration solution to give if your child has diarrhea. You should also take your child into Lwak clinic, or another clinic, to be seen by a clinical officer or nurse. If you are in a village that does not get zinc in the home, you should treat your child for diarrhea as you usually do at home and take him or her into the clinic where he/she will be seen by a clinical officer or nurse. Your child will receive zinc and ORS in the clinic. The way we will decide if your village gets zinc at home or not will be random, like flipping a coin in the air. We will pick some villages to get zinc at home and some to not get zinc at home for a year. Children in all villages will receive oral rehydration solution, called ORS, to prevent dehydration. Besides getting zinc, children with diarrhea in all villages will be the same in terms of visits to the house, care in the clinic and treatments for diarrhea.

The zinc pill can be dissolved in clean water or breast milk. The field worker will also give children in all villages in the study a few packets of ORS when they have diarrhea. ORS is important to stop your child from getting dehydrated. If your child is very sick at the time of the visit, the field worker will suggest that you take your child to the clinic to see the clinical officer. The study will make sure that the clinic always has drugs for your child. If your child has diarrhea, the field worker will visit your child 7 days later to see how he or she is doing.

If your child is too sick to be treated in the clinic, s/he will be referred to the district hospital. If your child has more than 2 weeks of diarrhea, s/he will also be referred to the district hospital.

For 165 children enrolled in the study, we want to collect less than a teaspoon of blood from your child’s finger tip that will be tested for zinc levels and anemia. These children will be selected at random like flipping a coin in the air. We are asking your permission to link the results of this testing with the other data from this study. Also we would like to link any information from clinic visits for your child, including malaria smear and culture of stool, to this study.

Your house will still be part of the DSS and the IEIP project. This means you will still be visited every 4 months as before to be asked about who lives in the house, new births, deaths, and people moving out or in. You will also be visited every two weeks as before to be asked about illnesses in the past two weeks. You can refuse to be part of this study and still be part of the DSS and IEIP. We are, however, asking your consent to use the facts collected from DSS and IEIP to link with the facts we collect for this study of zinc. *(Note that only children in Lwak will be part of the DSS).*

Benefit from being in this study:

If you are in a home zinc village, you will receive zinc and ORS at home and if you are in a village that does not get zinc at home, you will get ORS at home. These drugs can help prevent your child from getting severe dehydration. You can continue to go to Lwak clinic to receive free medical care as part of IEIP.

Risks from being in this study:

Zinc can rarely cause some side effects. The most common is vomiting, especially after taking zinc for the first time. Zinc has not been shown to cause severe side effects. ORS to treat diarrhea has no side effects. Blood draws from the finger tip can cause pain and brief bleeding.

You are free to choose to be part of this study. You have the right to refuse. If you do not want to go on with this study, you can stop at any time. If you stop the study, your child will still be able to get care at the clinic as part of IEIP. If you stop the study, your family will still be part of the DSS and IEIP, if you are part of it already.

The facts we collect in the study will be kept private to as much as allowed by law. No names will be used on any of the study reports. If you have any problems from the study and want to talk about it with someone who is not part of this study, you can contact Dr. Margaret Oloo at Aga Khan Hospital in Kisumu. Her phone number is 057 - 2021315. If you feel like you or your family might have been harmed by being in the study, or if you want to quit the study, please contact SUPERVISOR’S NAME or Dr. Daniel Feikin at the CDC office in Kisian (057-2022983).

Consent agreement:

| The consent form has been explained to me and I agree for my child to take part in the study. |
| --- |
|

| Child’s caretaker | Name: | Signature: | date□□/□□/□□ |
| --- | --- | --- | --- |
| Witness* | Name: | Signature: | date□□/□□/□□ |

* Subject may sign or provide verbal consent in the presence of a witness. The witness (by his/her signature) verifies that the consent form has been accurately translated to the subject and this is the subject’s signature or that he/she has provided verbal consent.

# Appendix B. Baseline Health survey of household

1. Date /./ 2. Interviewer code 

3a. First Name 3b. Juok Name 3c. Last (Father’s) name

4. Village ID  5. Compound ID  6. House ID 

7. DSS Permanent ID Number of child. --- (Use temporary permanent ID if child does not yet have a permanent ID, which should be village, compound, house and “99” for individual ID number. )

8. Date of birth  /  /  (dd/mm/yy) *(put 9’s if don’t know)*

9. Age (years/months/[days])  /  / [] (if child < 1 month old, record number days)

10. Sex Male Female

11a. In the last month, have you taken your child to a dispensary or health clinic? Yes No DK

b. If yes, how many times? _______

12a. In the last month, has your child been admitted overnight to a hospital? Yes No

12b. Reason for admission? (mark all that apply) Malaria Pneumonia Diarrhea other____________

13a. In the last month, has your child had diarrhea? Yes No Don’t Know

13b. If yes, did you seek health care outside of the home for the episode of diarrhea? Yes No DK

13c. If yes, which of the following places did you seek health care? (mark all that apply)

| Place visited |
| --- |
|  MOH Health center or dispensary |
|  Private Health center or dispensary |
|  Hospital |
|  Pharmacy/chemist |
|  duka/drug seller |
|  Community health worker |
|  Traditional healer/Spiritual healer |
|  Other |

13d. How did you treat your child for the episode of diarrhea? (mark all that apply)

| Treatment for diarrhea |
| --- |
|  Nothing |
|  Increased normal fluids and drinks, including breastfeeding |
|  Oral rehydration solution (ORS) |
|  antibiotic |
|  zinc |
|  Traditional medicine |
|  Other |

14. Have you ever given your child oral rehydration solution for diarrhea? Yes No Don’t Know

1. What do you use to treat diarrhea in your children? *(circle all that apply)*

| Treatment for diarrhea |
| --- |
|  Nothing |
|  Increased normal fluids and drinks, including breastfeeding |
|  Oral rehydration solution |
|  antibiotic |
|  zinc |
|  Traditional medicine |
|  Other |

16. Have you given your child vitamins in the last month? Yes No Don’t Know

17. Have you ever given your child zinc? Yes No Don’t know

18. Have you ever heard of zinc? Yes No Don’t Know

19a. Did your child sleep under a bednet last night? Yes No Don’t Know

19b. If yes, when was the bednet last treated with insecticide?

<= 9 months >9 months never been treated

20. Which of the following items do you have in your household?

Bicycle

Radio

Television

1. What is the roof of the house where the child sleeps made of?

Thatch/grass

Alumininum/tin

Concrete/brick

Other

1. what is the wall of the house where the child sleeps made of?

mud

cement covered mud

block/ fired brick/ stone

wood

tin

1. What kind of windows does where the child sleeps have?

None

Openings with temporary covers

Covered with wood/reeds

Covered with glass/screen

23 What do you use for calls?

Latrine

Bush

Cat Method

Other__________

*If child is less than 5 years of age, copy vaccination date for each vaccine from the card. Put ‘44’ in day column in card shows that a vaccination was given, but no date is recorded.*

24. Vaccination card available Yes No Don’t know

25. If card unavailable, why?  Lost it  It is someplace else  Never had it  Child never vaccinated  Other _____

*If card unavailable and child was vaccinated, ask mother to recall the vaccination dates the best she can and fill out table below. Prompt her with site of vaccination.*

26 Day Month Year

BCG *(left arm, leaves scar)*   

DTP/[Hep B/Hib], 1^st^ dose *(leg)*   

DTP/[Hep B/Hib], 2^nd^ dose *(leg)*    

DTP//[Hep B/Hib], 3^rd^ dose *(leg)*   

Oral polio, birth dose (OPV 0) *(drops in mouth)*   

Oral polio, 1st dose (OPV 1) *(drops in mouth)*   

Oral polio, 2nd dose (OPV 2) *(drops in mouth)*   

Oral polio, 3rd dose (OPV 3) *(drops in mouth)*   

Measles *(right arm)*   

Vitamin A (age at most recent) age in months _______

**Baseline measurements.**

27. Weight ____ Kg 27. Height ____ cm

28. Malaria smear  Positive  Negative  Not Done

28b. If positive, malaria parasites per 500 wbc _____

1. Hemoglobin ____gm/dl  Not done
2. Blood drawn for zinc level  Yes  No  Not done

**Appendix C. Household Morbidity Form** Interviewer code:______

Child’s first/Christian name _______________________ Juok/middle name___________________________ Last/father’s name _____________________

DSS ID number: . --- *(Use temporary permanent ID if child does not yet have a permanent ID, which should be village, compound, house and “99” for individual ID number. )*

*(Put 1 in days in which symptom was present, 0 if symptom not present, 9 if don’t know). Ask about every symptom. On the day of visit measure temperature and respiratory rate and evaluate for dehydration if current diarrhea, and chest indrawing and grunting if cough or difficulty breathing.*

Month:______________________

| *Symptom* | *If yes to symptom* | *1* | *2* | *3* | *4* | *5* | *6* | *7* | *8* | *9* | *10* | *11* | *12* | *13* | *14* | *15* | *16* | *17* | *18* | *19* | *20* | *21* | *22* | *23* | *24* | *25* | *26* | *27* | *28* | *29* | *30* | *31* |
| --- | --- | --- | --- | --- | --- | --- | --- | --- | --- | --- | --- | --- | --- | --- | --- | --- | --- | --- | --- | --- | --- | --- | --- | --- | --- | --- | --- | --- | --- | --- | --- | --- |
| *Diarrhea* |  |  |  |  |  |  |  |  |  |  |  |  |  |  |  |  |  |  |  |  |  |  |  |  |  |  |  |  |  |  |  |  |
|  | *# stools* |  |  |  |  |  |  |  |  |  |  |  |  |  |  |  |  |  |  |  |  |  |  |  |  |  |  |  |  |  |  |  |
|  | *Bloody?* |  |  |  |  |  |  |  |  |  |  |  |  |  |  |  |  |  |  |  |  |  |  |  |  |  |  |  |  |  |  |  |
| *Vomitting* |  |  |  |  |  |  |  |  |  |  |  |  |  |  |  |  |  |  |  |  |  |  |  |  |  |  |  |  |  |  |  |  |
|  | Everything? |  |  |  |  |  |  |  |  |  |  |  |  |  |  |  |  |  |  |  |  |  |  |  |  |  |  |  |  |  |  |  |
| *Unable to drink/breastfd* |  |  |  |  |  |  |  |  |  |  |  |  |  |  |  |  |  |  |  |  |  |  |  |  |  |  |  |  |  |  |  |  |
| *Fever* |  |  |  |  |  |  |  |  |  |  |  |  |  |  |  |  |  |  |  |  |  |  |  |  |  |  |  |  |  |  |  |  |
| *Cough* |  |  |  |  |  |  |  |  |  |  |  |  |  |  |  |  |  |  |  |  |  |  |  |  |  |  |  |  |  |  |  |  |
| *Difficulty breathing/fast breathing* |  |  |  |  |  |  |  |  |  |  |  |  |  |  |  |  |  |  |  |  |  |  |  |  |  |  |  |  |  |  |  |  |
| *Noisy breathing* |  |  |  |  |  |  |  |  |  |  |  |  |  |  |  |  |  |  |  |  |  |  |  |  |  |  |  |  |  |  |  |  |
| *Convulsions* |  |  |  |  |  |  |  |  |  |  |  |  |  |  |  |  |  |  |  |  |  |  |  |  |  |  |  |  |  |  |  |  |
| *Unresponsive/*  *lethargic* |  |  |  |  |  |  |  |  |  |  |  |  |  |  |  |  |  |  |  |  |  |  |  |  |  |  |  |  |  |  |  |  |
|  |  |  |  |  |  |  |  |  |  |  |  |  |  |  |  |  |  |  |  |  |  |  |  |  |  |  |  |  |  |  |  |  |
| *Visit Akala* |  |  |  |  |  |  |  |  |  |  |  |  |  |  |  |  |  |  |  |  |  |  |  |  |  |  |  |  |  |  |  |  |
| *Visit other clinic* |  |  |  |  |  |  |  |  |  |  |  |  |  |  |  |  |  |  |  |  |  |  |  |  |  |  |  |  |  |  |  |  |
|  |  |  |  |  |  |  |  |  |  |  |  |  |  |  |  |  |  |  |  |  |  |  |  |  |  |  |  |  |  |  |  |  |
| ***Day of visit*** |  |  |  |  |  |  |  |  |  |  |  |  |  |  |  |  |  |  |  |  |  |  |  |  |  |  |  |  |  |  |  |  |
| *Restless/irritable* |  |  |  |  |  |  |  |  |  |  |  |  |  |  |  |  |  |  |  |  |  |  |  |  |  |  |  |  |  |  |  |  |
| *Lethargic/unresponsive* |  |  |  |  |  |  |  |  |  |  |  |  |  |  |  |  |  |  |  |  |  |  |  |  |  |  |  |  |  |  |  |  |
| *Dehydration?* |  |  |  |  |  |  |  |  |  |  |  |  |  |  |  |  |  |  |  |  |  |  |  |  |  |  |  |  |  |  |  |  |
| *chest indraw?* |  |  |  |  |  |  |  |  |  |  |  |  |  |  |  |  |  |  |  |  |  |  |  |  |  |  |  |  |  |  |  |  |
| *grunting?* |  |  |  |  |  |  |  |  |  |  |  |  |  |  |  |  |  |  |  |  |  |  |  |  |  |  |  |  |  |  |  |  |
| *Temperature* |  |  |  |  |  |  |  |  |  |  |  |  |  |  |  |  |  |  |  |  |  |  |  |  |  |  |  |  |  |  |  |  |
| *Respiratory rate* |  |  |  |  |  |  |  |  |  |  |  |  |  |  |  |  |  |  |  |  |  |  |  |  |  |  |  |  |  |  |  |  |

**Appendix D. Diarrhea follow-up visit questionnaire (Use one form for follow-up of each diarrheal episode)**

Interviewer code 

Child’s First/Christian Name Juok/middle Name Last/Father’s name

DSS Permanent ID Number. (Use temporary ID if child does not yet have an ID, which should be village, compound, house and “99” for individual ID number. )

**Month:** ____________*(Put 1 in days in which symptom was present, 0 if symptom not present, 9 if don’t know; in row called Followup visit, put 3, 7, or 14 in day of f/u visit)*

| Symptom | 1 | 2 | 3 | 4 | 5 | 6 | 7 | 8 | 9 | 10 | 11 | 12 | 13 | 14 | 15 | 16 | 17 | 18 | 19 | 20 | 21 | 22 | 23 | 24 | 25 | 26 | 27 | 28 | 29 | 30 | 31 |  |
| --- | --- | --- | --- | --- | --- | --- | --- | --- | --- | --- | --- | --- | --- | --- | --- | --- | --- | --- | --- | --- | --- | --- | --- | --- | --- | --- | --- | --- | --- | --- | --- | --- |
| First day diarrhea |  |  |  |  |  |  |  |  |  |  |  |  |  |  |  |  |  |  |  |  |  |  |  |  |  |  |  |  |  |  |  |  |
| Day of first home visit |  |  |  |  |  |  |  |  |  |  |  |  |  |  |  |  |  |  |  |  |  |  |  |  |  |  |  |  |  |  |  |  |
| Follow up visit |  |  |  |  |  |  |  |  |  |  |  |  |  |  |  |  |  |  |  |  |  |  |  |  |  |  |  |  |  |  |  |  |
| Took zinc |  |  |  |  |  |  |  |  |  |  |  |  |  |  |  |  |  |  |  |  |  |  |  |  |  |  |  |  |  |  |  |  |
| Vomit in hour  after zinc |  |  |  |  |  |  |  |  |  |  |  |  |  |  |  |  |  |  |  |  |  |  |  |  |  |  |  |  |  |  |  |  |
| Took ORS |  |  |  |  |  |  |  |  |  |  |  |  |  |  |  |  |  |  |  |  |  |  |  |  |  |  |  |  |  |  |  |  |
| other meds* |  |  |  |  |  |  |  |  |  |  |  |  |  |  |  |  |  |  |  |  |  |  |  |  |  |  |  |  |  |  |  |  |
| Diarrhea |  |  |  |  |  |  |  |  |  |  |  |  |  |  |  |  |  |  |  |  |  |  |  |  |  |  |  |  |  |  |  |  |
| Vomiting/  spit up |  |  |  |  |  |  |  |  |  |  |  |  |  |  |  |  |  |  |  |  |  |  |  |  |  |  |  |  |  |  |  |  |
| Not eat/drink |  |  |  |  |  |  |  |  |  |  |  |  |  |  |  |  |  |  |  |  |  |  |  |  |  |  |  |  |  |  |  |  |
| Fever |  |  |  |  |  |  |  |  |  |  |  |  |  |  |  |  |  |  |  |  |  |  |  |  |  |  |  |  |  |  |  |  |
| Convulsions |  |  |  |  |  |  |  |  |  |  |  |  |  |  |  |  |  |  |  |  |  |  |  |  |  |  |  |  |  |  |  |  |
| Rash |  |  |  |  |  |  |  |  |  |  |  |  |  |  |  |  |  |  |  |  |  |  |  |  |  |  |  |  |  |  |  |  |
| Irritable/restless |  |  |  |  |  |  |  |  |  |  |  |  |  |  |  |  |  |  |  |  |  |  |  |  |  |  |  |  |  |  |  |  |
| Went to Lwak |  |  |  |  |  |  |  |  |  |  |  |  |  |  |  |  |  |  |  |  |  |  |  |  |  |  |  |  |  |  |  |  |
| Went to other clinic/hospital |  |  |  |  |  |  |  |  |  |  |  |  |  |  |  |  |  |  |  |  |  |  |  |  |  |  |  |  |  |  |  |  |
|  |  |  |  |  |  |  |  |  |  |  |  |  |  |  |  |  |  |  |  |  |  |  |  |  |  |  |  |  |  |  |  |  |
| *Day of visit* |  |  |  |  |  |  |  |  |  |  |  |  |  |  |  |  |  |  |  |  |  |  |  |  |  |  |  |  |  |  |  |  |
| Lethargic/  Unresponsive |  |  |  |  |  |  |  |  |  |  |  |  |  |  |  |  |  |  |  |  |  |  |  |  |  |  |  |  |  |  |  |  |
| Unable to drink |  |  |  |  |  |  |  |  |  |  |  |  |  |  |  |  |  |  |  |  |  |  |  |  |  |  |  |  |  |  |  |  |
| Sunken eyes |  |  |  |  |  |  |  |  |  |  |  |  |  |  |  |  |  |  |  |  |  |  |  |  |  |  |  |  |  |  |  |  |
| Restless/irritable |  |  |  |  |  |  |  |  |  |  |  |  |  |  |  |  |  |  |  |  |  |  |  |  |  |  |  |  |  |  |  |  |
| Skin pinch slow |  |  |  |  |  |  |  |  |  |  |  |  |  |  |  |  |  |  |  |  |  |  |  |  |  |  |  |  |  |  |  |  |
| Temperature |  |  |  |  |  |  |  |  |  |  |  |  |  |  |  |  |  |  |  |  |  |  |  |  |  |  |  |  |  |  |  |  |
| Refer to clinic? |  |  |  |  |  |  |  |  |  |  |  |  |  |  |  |  |  |  |  |  |  |  |  |  |  |  |  |  |  |  |  |  |

*Name(s) of other meds taken for diarrhea. *(observe in home if possible)*  Panadol  Cotrimoxazole  Nalidixic acid  Other ______

**Appendix E. Clinic Sick Visit questionnaire**

**Part 1: Identifying information**

Date  / / 

Child’s First/Christian Name Juok/middle Name Last/Father’s name

DSS Permanent ID Number.  Child’s age in months 

What is relationship of the caregiver to the child? ?  Self  Mother  sister  father  grandmother  aunt  adopted mother/guardian  Other ­­­­­­­­­­­­­­­­­­­­­­­­­­­­­­­

**Part 2: History and examination (bolded questions are part of exam)**

*Vital Signs*

Pulse  (per 1 minute) Respiratory rate  (per one minute) Temperature . ^o^ C

*Danger signs*

Is the child unable to drink or breastfeed at all?  Yes  No

Does the child vomit everything with each feeding?  Yes  No

Is the child lethargic or unconscious? (by history)  Yes  No

**Is the child lethargic or unconscious? (by observation)  Yes  No**

Has the child had convulsions with this illness?  Yes  No

IF THE CHILD HAS ANY OF THE ABOVE, THEY HAVE A DANGER SIGN. THEY WILL LIKELY NEED REFERRAL.

*Malaria*

Has the child had fever or hot body?  Yes  No

Has the child had midhusi?  Yes  No

Did the child sleep under a bednet last night?  Yes  No

If yes, when was it last treated?  <9 months  > 9 months  never

*ARI*

Has the child had a cough?  Yes  No

Has the child had difficulty breathing?  Yes  No

**Does the child have chest indrawing?  Yes  No**

**Does the child have stridor/grunting while calm?  Yes  No**

**Does the child have wheezing?  Yes  No**

**Does the child have nasal flaring?  Yes  No**

*Diarrhea*

Has the child had diarrhea?  Yes  No

How many days of diarrhea? 

What was maximum number of stools in 24 hours? 

Has there been bloody diarrhea?  Yes  No

Has the child had any vomiting?  Yes  No

**Is the child restless or irritable?  Yes  No**

**Does the child have sunken eyes?  Yes  No**

**Is the child not able to drink or drinking poorly?  Yes  No**

**Is the child drinking eagerly? Is thirsty?  Yes  No**

**After pinching the skin does it go back slowly?  Yes  No**

*Others*

**Does the child have a stiff neck?  Yes  No**

**Does the child have a bulging fontanelle?  Yes  No**

**Does the child have a rash?  Yes  No**

**Is a localized rash or a diaper rash?  Yes  No**

**Does the child have mouth ulcers?**   Yes  No

Does the child had ear pain or discharge?  Yes  No

**Is there pus draining from ear?  Yes  No**

**Is there tender swelling behind ear?  Yes  No**

**Any signs of paleness in child (palms, nailbed, mucosa)  Yes  No**

Does the child have yellow eyes?  Yes  No

Has the child lost weight with this illness?  Yes  No

**Part 3: Diagnosis**

Malaria  Anemia 

Cerebral malaria  Malnutrition 

Pneumonia  Meningitis 

URTI  Measles 

Otitis media  Convulsions 

Wheezing/asthma  Rash/skin problem 

Diarrhea/gastroenteritis  Eye problem 

Dehydration  Wound/Injury 

Dystentary  Burn 

Worms  Anaphylaxis 

Other ____________________________-

Was the child referred to seek hospital care?  Yes  No

**Part 4: Treatment**

Analgesics (Panadol, Aspirin) 

Amodiaquine 

Coartem (lumafantrin/aretmether 

Quinine 

SP 

Septrin (cotrimoxazole) 

Penicillin V 

Amoxacillin 

Gentamicin 

Nalidixic acid 

Ciprofloxacin 

Cloxacillin 

Tetracycline 

Antihelminthic (mebendazole/albendazole) 

ORS 

Salbutamol 

Piriton (chloropheniramine, antihistamine) 

Other: ___________________________

**Part 5: Samples**

Was a malaria blood smear done?  Yes  No

*If the child has fever, malaria, or diarrhea, they should have a malaria blood smear:*

Was a stool sample taken for etiologic testing?  Yes  No

**Part 6: Adverse Events**

*To be filled out by the clinical officer:*

Is this clinic visit due to an unanticipated adverse event due to being in the zinc study?  Yes  No  Not sure

*Unanticipated adverse events include: anaphylaxis, severe rash or mucocutaneous reaction, continuous vomiting, sudden unexpected death without a clear cause in a previously well child, injury due to study procedure or participation.*

IF YES, MUST COMPLETE AN ADVERSE FORM AND REPORT TO PI.

**Appendix F. Incident Investigation Form**

Child’s first/Christian name _______________________ Juok/middle name___________________________ Last/father’s name _____________________

DSS ID number: . --- *(Use temporary permanent ID if child does not yet have a permanent ID, which should be village, compound, house and “99” for individual ID number. )*

Date of Birth . // Sex  Male  Female

Notification of INCIDENT was received from whom?

 Field worker  Clinical Officer  Other Akala clinic staff  Field supervisor  DSS staff  Siaya DH staff

 PGH staff  Other _____________________________

Onset date // Date of awareness by study staff //

Type of incident *(check all that apply)*

 Hospitalization  Death  Anaphylaxis/severe allergy  Rash/Mucocutaneous reaction

 Injury due to study participation  Breach of protocol  Other ________________________

Date that INCIDENT began : // Time INCIDENT began: : *(use 24 hour clock)*

Did the INCIDENT occur within 1 week of taking zinc?  Yes  No

Date last zinc taken: // Time last zinc taken: : *(use 24 hour clock)*

How many days of zinc did the child take before INCIDENT occurred? 

**Describe in detail the nature & timing of the INCIDENT (attach addendum if necessary):**

At the time of completing this report, determine status of INCIDENT?  Resolved  Not resolved  N/A

If event is resolved, determine status?  died  disability  resolved without disability  N/A

 Other ________________________________

If event is not resolved, determine status?  Hospitalized  participant home, event ongoing  N/A

 Other_____________________________

Part 3. Categorization/Rating *(To be completed by principal investigator)*

The likelihood that the INCIDENT was caused by the study is (definitions given at the end of form):

Definitely (direct association with research intervention)

Probably (more likely explained by research intervention than other cause)

Possibly (research intervention and other cause explain incident equally well)

Probably not (more likely explained by other cause than research intervention)

Not (clearly explained by another, documented cause)

CDC/IRB categorization of a reportable incident:

Unanticipated problems involving risks to subjects or others

Including serious, unexpected, and possibly related adverse events

Other unanticipated problems involving risks to subjects

Breaches of protocol and other examples of noncompliance with protocol or IRB requirements

Premature suspension or termination of study

Other incident defined as reportable in protocol or requested by IRB

Incident not reportable

Should the participant be withdrawn from study?  Yes  No

Has form 1254 been sent to CDC IRB?  Yes  No  N/A

_____________________ ___________

Signature of investigator Date

**Appendix G. Incident report form (0.1245)**

## Incident Report

Office of Scientific Regulatory Services

Please report incidents to HRPO by e-mail within 2 working days of CDC’s awareness, to be followed by a completed form 0.1254 within 2 weeks of CDC’s awareness. Supplementary information may be submitted at any time. Incident reports receive highest priority at HRPO.

| For HRPO use only | | | | | | | Date Received | | | | Protocol # | |
| --- | --- | --- | --- | --- | --- | --- | --- | --- | --- | --- | --- | --- |
| Protocol Title: | | | | | | | | | | | | |
| Key CDC Personnel | | | | | | | | | | | | |
| Principal investigator None    Primary contact    Person completing report (if different) | | | User ID | SEV # | | | | CIO/Division | | | | |
| Characterization of Incident | | | | | | | | | | | | |
| This report covers (check all that apply) ^103(b)(5)^  unanticipated problem(s) involving risks to subjects or others  serious, unexpected, possibly related adverse event(s)   [include supplemental information, such as on form 0.1254S]  other unanticipated problem(s) involving risks to subjects or others  serious or continuing noncompliance with regulations or IRB requirements (e.g., breach of protocol)  suspension or termination (for reasons other than expiration) [if termination, also submit 0.1253]  other incidents as specified in protocol or requested by IRB  [if adverse event, include supplemental information, such as on form 0.1254S]  Recurrence:  One or more incidents have been previously reported for this protocol. | | | | | | | | | | | | |
| Timing of Incident and Awareness | | | | | | | | | | | | |
|  | | | | | | Begin date | | | End date (if range) | | | |
| Date(s) that incident(s) occurred | | | | | |  | | |  | | | |
| Date(s) that site became aware of incident(s) | | | | | |  | | |  | | | |
| Date(s) that CDC became aware of incident(s) | | | | | |  | | |  | | | |
| Further explanation of timing as needed: | | | | | | | | | | | | |
| Site Information | | | | | | | | | | | | |
| Name, location, funding information, and assurance number of institution where research was conducted | | | | | | | | | | | | |
| Funding Award | | Institution | | | Location | | | | | OHRP Assurance Number | |  |
| Mechanism | Number |  |
|  |  |  | | |  | | | | |  | |  |
| Name of principal investigator at site of occurrence    Title of protocol at site (if different from CDC title)    Tracking number of protocol at site | | | | | | | | | | | | |
| Description of Incident | | | | | | | | | | | | |
| Include study identifiers of affected subjects as appropriate. Describe current status, changes in status, and resolution, as appropriate. If this incident is an adverse event, please also describe seriousness, expectedness, and relationship with study intervention.    This incident changes the harm-benefit profile of the research. | | | | | | | | | | | | |
| Actions Taken to Address Incident | | | | | | | | | | | | |
| Explain all actions taken, including revisions of protocol or consent process.    The protocol has been or needs to be revised [also submit Form 0.1252]  The consent process or document has been or needs to be revised [also submit Form 0.1252] | | | | | | | | | | | | |
| Other Entities to Whom this Incident Is Being Reported (e.g., local IRB, DMC, US and foreign regulators) | | | | | | | | | | | | |
| List entities to whom incident is being reported. Indicate briefly whether there is regular monitoring, the kind of expertise in monitoring, and the intensity of review. | | | | | | | | | | | | |

**Appendix H. Adverse Event form – supplement to incident report**

## Adverse Event Report—Supplement to Incident Report

Office of Scientific Regulatory Services

Form 0.1254S, or any other format containing the same information, may be used to supplement an Incident Report submitted on Form 0.1254 when reporting an adverse event. Among incidents classified as adverse events, CDC procedures require reporting of only those that are deemed serious, unexpected, and possibly related to the research procedures. The protocol or IRB might require other adverse events to be reported as well. Please see the instructions for Form 0.1254 for additional information.

| For HRPO use only | | | | | | Date Received | | | | Protocol # | | | |
| --- | --- | --- | --- | --- | --- | --- | --- | --- | --- | --- | --- | --- | --- |
| Protocol Title: | | | | | | | | | | | | | |
| Key CDC Personnel | | | | | | | | | | | | | |
| Principal investigator None    Primary contact | | | User ID | | SEV # | | | | CIO/Division | | | | |
| Timing of Incident and Awareness | | | | | | | | | | | | | |
|  | | | | | | | | Begin date | | | End date (if range) | | |
| Date(s) that incident(s) occurred | | | | | | | |  | | |  | | |
| Date(s) that site became aware of incident(s) | | | | | | | |  | | |  | | |
| Date(s) that CDC became aware of incident(s) | | | | | | | |  | | |  | | |
| Further explanation of timing as needed:    This is a follow-up report on a previously reported adverse event. | | | | | | | | | | | | | |
| Site Information | | | | | | | | | | | | | |
| Name, location, funding information, and assurance number of institution where research was conducted | | | | | | | | | | | | | |
| Funding Award | | Institution | | | | | Location | | | | | OHRP Assurance Number |  |
| Mechanism | Number |  |
|  |  |  | | | | |  | | | | |  |  |
| Name of principal investigator at site of occurrence | | | | | | | | | | | | | |
| Details of Person Reporting Adverse Event | | | | | | | | | | | | | |
| Name, qualifications, and contact information of person reporting adverse event | | | | | | | | | | | | | |
| Sponsor/Company Details (funder or provider of interventional product) | | | | | | | | | | | | | |
| Name and address of sponsor/manufacturer/company    Name and contact information of contact person in reporting company or institution    Identifying regulatory code or number for the suspected product    Sponsor/manufacturer’s identification number for the case (this number must be the same for the initial and follow-up reports on the same case). | | | | | | | | | | | | | |
| Participant Details | | | | | | | | | | | | | |
| Study identifier, sex/gender, ethnicity, race, age, detainment (if applicable),  anthropometrics, condition, potentially relevant medical history | | | | | | | | | | | | | |
| Suspected Intervention (e.g., medicinal products) | | | | | | | | | | | | | |
| Name(s), indication(s), dosage/route, start/stop/duration of exposure | | | | | | | | | | | | | |
| Other Interventions | | | | | | | | | | | | | |
| Name(s), indication(s), dosage/route, start/stop/duration of exposure | | | | | | | | | | | | | |
| Description of Adverse Event | | | | | | | | | | | | | |
| Signs, symptoms, diagnosis    Start/stop/duration of event    Dechallenge/rechallenge information    Outcome: recovery, sequelae, death; diagnostics; cause of death | | | | | | | | | | | | | |
| Analysis of Adverse Event | | | | | | | | | | | | | |
| Severity (check one)  5 Death  4 Life-threatening at time of event  3 Severe  2 Moderate  1 Mild  0 Normal  Additional information (check all that apply)  Required hospitalization or prolongation of existing hospitalization  Resulted in persistent or significant disability or incapacity  Is a congenital anomaly or birth defect | | | | Explain: | | | | | | | | | |
| Expectedness  0 Event expected  1 Event was unexpected  Event not previously documented with this intervention  Severity greater than previously documented with this intervention  Frequency greater than previously documented with this intervention | | | | Explain: | | | | | | | | | |
| Relationship event to study intervention  5 Definitely related  4 Probably related  3 Possibly related  2 Probably not related / unlikely  1 Not related / unrelated (specify documented alternative cause) | | | | Explain: | | | | | | | | | |

**Appendix I. Curriculum vitae for non-KEMRI employees**

CVs for Feikin, Breiman, Hamel, and Slutsker are already on file with SSC.

Appendix J. **IEIP Morbidity Surveillance Study**

**Clinic form for Zinc-Sera Assays – Children < 5 years old**

(Prefilled demographic info)

Name of the child: __________________________

First Name: ____________________________

Juok Name: ____________________________

Last Name: ____________________________

Child’s Date of Birth: __ / __ / ____

Child’s DSS Permanent ID: _______________________

Child’s Temporary DSS ID: _______________________

Village Name: _________________________

Compound Head Name: _______________________

Mother’s Name: _________________________

Date sample obtained: __ / __ / ____ Demographics filled by: __

Has the child taken any zinc tablets yet? Yes / No

Was sample obtained? Yes / No

Sample Label:

Appendix K. **IEIP Morbidity Surveillance Study**

**Zinc Study – Enrollment Form – Children < 5 years old**

(Prefilled demographic info.)

Name of the child: __________________________

First Name: ____________________________

Juok Name: ____________________________

Last Name: ____________________________

Child’s Date of Birth: __ / __ / ____

Child’s DSS Permanent ID: _______________________

Child’s Temporary DSS ID: _______________________

Village Name: _________________________

Compound Head Name: _______________________

Mother’s Name: _________________________

Date of visit: __ / __ / ____ Demographics filled by: __

Number of visits or attempts: 1 / 2 / 3 / Other: ____________

Enrolled? accepted / rejected / unavailable / Other: ________

Selected for blood-zinc level? Yes / No

Agrees to have blood drawn? Yes / No

**Appendix L. IEIP Morbidity Surveillance Study - Zinc Study VR’s Home-Visit Survey**

Date of Interview: __ / __ / ____ Demographics Filled by: ____

First Name / Juok Name / Last Name / Date of Birth

Child’s DSS Permanent ID / *Namba nyathi mar DSS*: _____________________

Village Name: ________________ Head of Compound: ______________________

Mother’s Name: ________________

1. Was Mother or Caretaker home today? Yes / No

(*If Mother or Caretaker not at home, don’t ask the following questions*)

2. Was Zinc given today? Yes / No

3. Were ORS packets given today? Yes / No

4. Which visit is this? First visit / Follow-up visit

(*If 1^st^ visit, skip rest of these questions, and continue to educate about diarrhea and Zinc/ORS Treatment*)

5. Did you use any of the zinc tablets since my last visit? Yes / No / Don’t Know

(if “no” skip to 12.)

6. Who was given the pills? Participant / another son or daughter /

*Nga kendo mane giyedhe modong*? Father or Mother / Don’t Know / Other

7. Why was the zinc given? Diarrhea / vomiting / fever / cough / difficulty breathing/ Other ________

8. Do you think the zinc helped or worked for the diarrhea? Yes / No / Don’t Know

*Bende iparo ni Zinc ne okonyo kata ne otiyo kuom diepni*? Ee / Ooyo

9. If yes, how do you think it helped? Decreased the amount of diarrhea /

(if “no” skip to 10.) Shortened the course of the episode /

Increased appetite / Other ________

10. Do you think the zinc helped any of the other symptoms? Yes / No / Don’t Know

*Bende iparo ni Zinc ne okonyo e tuoche mamoko*? Ee / Ooyo

11. If yes, which symptoms? / *Ka ee, to mage*? Diarrhea / vomiting /

fever / cough / difficulty breathing/ Other: ___________

*Ask caretaker to show you the zinc packet(s)*

12. Do you still have the zinc packet available for me to see? Yes / No

13. How many tablets were used from the last blister pack?

*Yedhe adi mane otigo e paket mogik*?        0/ 1/ 2/ 3/ 4/ 5/ 6/ 7/ 8/ 9/ 10 / N/A
